# Integrated Approach for the Discovery of Antifungal and Antibiofilm Agents From Cerrado Plants

**DOI:** 10.1002/cbdv.71532

**Published:** 2026-07-26

**Authors:** Lorena C. Albernaz, Amanda M. Almeida, Laís S. Morais, Alice M. S. Rodrigues, Rodrigo S. Ferreira, Laila S. Espindola, Marion Girardot, Christine Imbert

**Affiliations:** ^1^ Laboratório de Farmacognosia Universidade de Brasília Brasília Brazil; ^2^ Laboratoire De Biodiversité Et Biotechnologies Microbiennes (LBBM), Observatoire Océanologique Université Sorbonne Banyuls‐sur‐Mer France; ^3^ Laboratoire Ecologie et Biologie des Interactions (EBI) Université De Poitiers Poitiers France

**Keywords:** antifungal agents, *Candida albicans*, Cerrado biome, molecular networking, yeast‐to‐hyphae transition

## Abstract

The increasing incidence of *Candida albicans* infections, especially those involving drug‐resistant strains, highlights the need for new antifungal agents. In this study, 108 plant extracts from native and endemic species of the Brazilian Cerrado were screened against *C. albicans* in both planktonic and biofilm forms (developing and mature). Eighteen extracts demonstrated significant antifungal and antibiofilm activity, particularly from species in the Fabaceae, Myrtaceae, and Celastraceae families. Extracts from *Hymenaea stigonocarpa* notably inhibited the yeast‐to‐hyphae transition, a key virulence factor. Using molecular networking (GNPS) and in silico tools (SIRIUS), 60 putative compounds were annotated, including xanthones, flavonoids and triterpenoids, with selected candidates showing favorable binding profiles in molecular docking analyses. This integrative metabolomic approach enabled the identification of bioactive scaffolds, reinforcing the Cerrado biome as a valuable source of structurally diverse metabolites for antifungal drug discovery.

## Graphical abstract

A comprehensive screening of 108 Brazilian Cerrado plant extracts identified potent inhibitors of *Candida albicans* biofilm formation. Active samples underwent UPLC–MS/MS and molecular networking for compound annotation, followed by in silico docking. This integrated workflow accelerates the discovery of bioactive metabolites by combining experimental screening with advanced computational analysis.

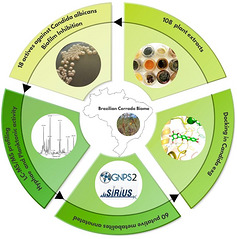

## 1 Introduction

The ever‐increasing incidence of *Candida albicans* infections, particularly those involving resistant strains, has intensified the search for novel antifungal agents. Indeed, 8 of the 37 antifungals introduced between 1981 and 2023 were derived from natural sources [1, 2].

Although *Candida* spp. are part of the normal human microbiota, they can cause infections under immunocompromised conditions [3, 4]. The rising incidence of antifungal resistance and the burden on healthcare systems make candidiasis a significant societal challenge. Approximately 80% of infections occurring in Intensive Care Units are caused by *Candida* spp. and are associated with a high risk of patient death, reaching 98% when treatment is delayed [5]. Although *C. albicans* is the most prevalent, nonalbicans species including *Candida parapsilosis*, *Candida tropicalis* and *Candida glabrata* account for 30%–54% of cases [6, 7, 8].

Bacteria and fungi can form biofilms, microbial communities adhered to biotic or abiotic surfaces embedded in a self‐produced extracellular matrix of polysaccharides, proteins, lipids, and extracellular DNA [9]. Biofilm formation poses a major therapeutic challenge by markedly increasing resistance of *Candida* spp. to conventional antifungals, including azoles and amphotericin B [10]. This resistance is associated with factors such as cell physiology within the biofilm, extracellular polymeric substances, efflux pump overexpression, and altered sterol content [11]. Natural products may function as antifungal agents by targeting alternative pathways such as inhibiting biofilm formation, disrupting cell walls, and inducing apoptosis [12, 13].

The *C. albicans* exo‐β‐1,3‐glucanase system is a compelling antifungal target due to its central role in masking immunogenic β‐glucan on the fungal cell surface. In *C. albicans*, exo‐1,3‐β‐glucanase (Exg) is a GH5 family enzyme responsible for cleaving and/or transferring glucose residues in cell wall β‐1,3‐glucans. It functions exolytically, removing units from the nonreducing end of the polymer, and has a role in transglycosylation, enabling the transfer of glucan fragments between chains. This activity is crucial for β‐D‐glucan remodeling, chain length regulation, and fungal morphogenesis, directly impacting *C. albicans* cell wall integrity. Furthermore, exo‐β‐1,3‐glucanase (Exg) is involved in cell wall remodeling and biofilm maturation, constituting a promising antifungal target [14].

Numerous plant extracts/compounds possess potent anti‐*Candida* properties [15, 16, 17], with a low number pertaining to biofilm assays. *Candida* biofilm formation and maturation is highly complex [18], involving a diverse population of cell types [19]. The Cerrado, Brazil's second largest biome and reservoir of chemical and biological diversity, faces constant threats from livestock expansion and agribusiness, further exacerbated by climate change and, recently, the criminal use of fire in politically driven contexts.

Since 1998, the Laboratory of Pharmacognosy at the University of Brasília, Brazil has established and populated the “Cerrado Plant Extract Bank”, which currently houses about 7000 plants and endophytic fungi samples [15, 20, 21, 22]. This collection constitutes a valuable resource for bioprospecting and offers strong potential for the discovery of antifungal compounds. Fabaceae, Myrtaceae and Celastraceae families are among the families found in the biome [15, 23, 24]. The aims of this study were to investigate 108 extracts from native Cerrado plants for antifungal and antibiofilm activity against *C. albicans*. An integrated approach involving high‐resolution mass spectrometry combined with molecular networking to correlate bioactivity with chemical profiles.

## 2 Results and Discussion

### 2.1 Screening Against *Candida albicans* Biofilm

Biofilm inhibition was initially assessed in a fixed‐concentration screening assay (250 µg mL^−1^). Extracts exhibiting ≥ 50% inhibition under these conditions were classified as active, following criteria commonly used in large‐scale antifungal screenings. These active samples were subsequently prioritized for LC–MS/MS profiling and further evaluated in assays against planktonic cells and hyphal transition. Of the 108 extracts tested, 18 met the activity cutoff for either biofilm development or mature biofilm formation (Table 1) and were therefore selected for further evaluation against 2 clinical isolates, S1 and S3. The results for the remaining 90 extracts are provided in Table SI1.

**TABLE 1:** Inhibition percentages (mean ± SD; 95% CI) of *Candida albicans* (ATCC 28367) biofilm formation and mature biofilms. Δ% change was calculated as the inhibition percentage of mature biofilms minus that of biofilm formation. p‐values indicate comparisons between developmental stages for the same extract using Welch's *t*‐test.

		% Inhibition ± SD (CI 95%)			
Sample	Plant part (solvent)	Biofilm in formation	Mature biofilm	Δ% change	*p*‐value
*Andira humilis*	RB (EA)	54.48 ± 6.28 (47.88‐61.08)	65.55 ± 12.23 (46.72‐67.17)	+11.07	ns
*Blepharocalyx salicifolius*	SW (EA)	62.09 ± 8.95 (52.68‐71.49)	39.76 ± 13.05 (26.58‐52.67)	−22.33	^*^
	SB (EA)	63.59 ± 16.06 (46.73‐80.45)	37.03 ± 19.75 (17.08‐57.01)	−26.56	^*^
*Cheiloclinium cognatum*	L (E)	59.28 ± 18.47 (39.89‐78.68)	45.82 ± 17.63 (28.01‐63.66)	−13.46	ns
*Hymenaea stigonocarpa*	SW (H)	61.58 ± 3.31 (58.10‐65.06)	60.94 ± 8.17 (52.69‐69.21)	−0.64	ns
	L (EA)	65.29 ± 14.24 (50.34‐80.24)	63.65 ± 12.27 (51.25‐76.07)	−1.64	ns
	L (H)	57.37 ± 19.65 (36.75‐78.00)	59.86 ± 4.41 (55.41‐64.34)	+2.49	ns
*Myrcia linearifolia*	R (EA)	40.66 ± 10.93 (29.19‐52.14)	64.38 ± 12.67 (51.58‐77.21)	+23.72	^**^
	AP (EA)	42.60 ± 13.38 (28.56‐56.64)	59.46 ± 11.88 (47.46‐71.48)	+16.86	^*^
*Sclerolobium aureum*	RW (H)	57.55 ± 7.05 (50.15‐64.96)	58.08 ± 6.25 (51.77‐64.41)	+0.53	ns
	RW (E)	69.12 ± 4.70 (64.18‐74.06)	72.07 ± 7.08 (64.92‐79.23)	+2.95	ns
	RW (EA)	55.93 ± 7.18 (48.38‐63.47)	75.22 ± 2.84 (72.35‐78.11)	+19.29	^**^
	SW (E)	61.80 ± 3.85 (57.76‐65.85)	63.34 ± 5.95 (57.33‐69.36)	+1.54	ns
	SW (EA)	61.24 ± 11.74 (48.91‐73.56)	62.86 ± 9.94 (52.82‐72.92)	+1.62	ns
	SB (EA)	55.90 ± 7.22 (48.31‐63.48)	64.31 ± 5.32 (58.94‐69.70)	+8.41	^*^
*Tachigali vulgaris*	SW (EA)	72.13 ± 3.82 (68.12‐76.14)	65.88 ± 10.61 (55.16‐76.61)	−6.25	ns
*Vatairea macrocarpa*	RW (EA)	67.59 ± 3.33 (64.09‐71.08)	77.19 ± 1.74 (75.43‐78.97)	+9.60	^**^
	L (EA)	62.01 ± 3.75 (58.05‐65.97)	73.31 ± 2.87 (70.42‐76.23)	+11.30	^**^

**Abbreviations**: AP, aerial parts; CI, confidence interval; E, ethanol; EA, ethyl acetate; H, hexane; L, leaves; ns, not significant; R, root; RB, root bark; RW, root wood; S, stem; SB, stem bark; SD, standard deviation; SW, stem wood.

* *p* < 0.05

** *p* < 0.01.

The extracts active against both biofilm stages were predominantly of medium polarity, with activity observed in 25% of ethyl acetate samples, 13% of ethanolic, and 9% of hexane extracts. This distribution is consistent with the chemical profiles of these solvents, since compounds commonly extracted with ethyl acetate, including terpenes (monoterpenes and sesquiterpenes), flavonoids and alkaloids, have been reported as effective against biofilms [25, 26, 27].

The Fabaceae family is renowned for biosynthesizing flavonoids which are recognized for their antimicrobial activity [15, 16, 28]. Among the extracts tested against the *C. albicans* (ATCC 28367) strain, the most active against both biofilm development and mature biofilm stages were the ethyl acetate extracts from the root wood of *Vatairea macrocarpa* and the stem wood of *Tachigali vulgaris*, with inhibition rates of at least 67.59% (biofilm development) and 65.88% (mature biofilm), respectively. Extracts from other Fabaceae species also demonstrated notable antibiofilm activity. Ethyl acetate extracts from *Sclerolobium aureum*, *Hymenaea stigonocarpa*, and *Andira humilis* inhibited biofilm formation by 55.93%, 57.37%, and 54.48%, respectively. Additionally, the hexane extracts from *Staphylococcus aureum* and *Hymenaea stigonocarpa*, along with the ethyl acetate extract from *Aloe humilis*, reduced mature biofilms by 58.08%, 59.86%, and 65.55%, respectively.

The Myrtaceae and Celastraceae families are cited for producing phenolic compounds and triterpenes, respectively, which possess antifungal properties [29, 30, 31]. In this study, ethyl acetate extracts from *Blepharocalyx salicifolius* stem bark and wood, as well as the ethanol extract from *Cheiloclinium cognatum* leaves, showed activity exclusively against developing *C. albicans* (ATCC 28367) biofilms, with minimum inhibition rates of 62.09% and 59.28%, respectively. The corresponding inhibition percentage against mature biofilms was < 46%. Conversely, ethyl acetate extracts from *Myrcia linealifolia* aerial parts and roots exhibited higher activity against mature biofilms (inhibition ≥ 59.46%) than developing biofilms (≥ 40.66%). To our knowledge, there are no previous reports on the antibiofilm activity of these species. Therefore, the antibiofilm activity of the 18 selected active extracts was further evaluated against two clinical *C. albicans* strains (S1 and S3).

### 2.2 Antibiofilm Activity Against *Candida albicans* Clinical Strains

**FIGURE 1:**
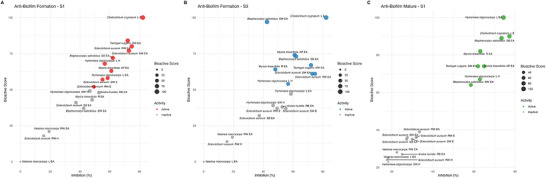
Inhibition of biofilm formation and mature biofilm by plant extracts. Scatterplot shows the relationship between the percentage inhibition of (A) biofilm formation (S1 clinical strain) and the bioactive score of each extract. Active extracts are shown in red; inactive extracts are shown in gray. (B) Biofilm formation (S3 clinical strain) versus bioactive score. Active extracts are shown in blue; inactive extracts are shown in gray. (C) Mature biofilm (S1) versus bioactive score. Active extracts are shown in green; inactive extracts are shown in gray. Scientific names are presented in italics. Bioactive scores correspond to the relative magnitude of antifungal activity according to pre‐established scoring criteria. AP: aerial parts; L, leaves; R, root; RB, root bark; RW, root wood; S, stem; SB, stem bark; SW, stem wood; H, hexane; EA, ethyl acetate; E, ethanol.

Among the 18 extracts that exhibiting anti‐development and/or anti‐biofilm activity against the *C. albicans* (ATCC 28367), approximately 66% also inhibited biofilm formation of the clinical isolates S1 or S3 by > 50%. However, only 39% of these extracts were active against both isolates simultaneously (Figure 1A–C; Tables SI2A, SI2B). The differences observed between the collection strains and clinical isolates emphasize the greater resistance of biofilms formed by patient‐derived *C. albicans* strains. It is noteworthy that the activity detected in our assays, despite this increased resistance, supports the potential of Cerrado extracts as antifungal development candidates. These findings are consistent with earlier studies reporting reduced susceptibility of clinical isolates [32, 33, 34].

Several Cerrado extracts demonstrated promising antibiofilm activity against both clinical isolates (*C. albicans* S1 and S3), notably the ethyl acetate from: *Barkleyanthus salicifolius* stem bark; *M. linealifolia* aerial parts; *S. aureum* stem/root, and *Thymus vulgaris* stem wood; together with the *C. cognatum* leaves ethanol extract and the *Hymenaea stigonocarpa* leaves hexane extract. These species belong to families rich in phenolic compounds and terpenes, which have been associated with anti‐*Candida* activity, particularly through disruption of planktonic cell adhesion and inhibition of biofilm metabolic activity [27, 35]. Consistent with their phytochemical profiles, Fabaceae and Myrtaceae species have been highlighted for their antifungal potential, including specific action against *Candida* biofilms [25, 26]. Meanwhile, a number of extracts demonstrated selective inhibition of the S1 clinical isolate only, including ethyl acetate extracts: *H. stigonocarpa* (leaves) and *A. humilis* (root bark), together with the *S. aureum* (stem wood) ethanol extract (Table SI2). In addition, ethyl acetate extracts from *B. salicifolius* (stem wood) and *M. linealifolia* (roots) were only active against the S3 clinical isolate. Collectively, these results reveal strain‐dependent variation in susceptibility and reinforce the antifungal potential of Cerrado plant extracts/compounds.

Notably, no extract achieved > 50% inhibition against the S3 mature biofilm under the tested conditions, suggesting lower susceptibility of this clinical isolate. This finding likely reflects the lower susceptibility of this clinical isolate, which may be associated with intrinsic factors such as increased biofilm structural complexity, higher extracellular polymeric substance (EPS) production, and/or enhanced expression of resistance‐related genes commonly observed in mature biofilms. These characteristics can limit the penetration and efficacy of bioactive compounds [9, 11, 32]. In contrast, 8 extracts inhibited the S1 mature biofilm, with inhibition rates ranging from 61.95% to 77.23%. Among these, 5 extracts also exhibited >60% inhibition against the ATCC strain mature biofilm and were therefore considered the most promising candidates: *H. stigonocarpa* leaves (hexane and ethyl acetate); *M. linearifolia* root and aerial parts (ethyl acetate), and *T. vulgaris* stem wood (ethyl acetate) (Table SI2).

Among these, the ethyl acetate *H. stigonocarpa* leaves extract was the most active, with a biofilm score (BS) of 100 against the S1 clinical isolate. Extracts from four Myrtaceae species extracts exhibited interesting activity in the mature biofilm assay (BS ranging from 64 to 89). Among other family extracts, the ethanol extract from *C. cognatum* leaves demonstrated significant activity (BS: 90). Sensitivity analysis confirmed the robustness of the BS cutoff. Agreement between BS ≥ 60 and BS ≥ 50 was 83%–100% (*κ* = 0.67–1.00), while BS ≥ 60 and BS ≥ 70 showed 77.8%–88.9% agreement (*κ* = 0.53–0.77) across datasets (Table SI3). These findings indicate that active extract classification is not strongly affected by small variations in threshold. Although correlations between BS values and conventional antifungal endpoints (MIC and hyphal inhibition) were weak (*ρ* between –0.32 and +0.21, *p* > 0.14), the extracts with high BS generally aligned with those selected for further biological evaluation, supporting its use as a prioritization tool in large‐scale screenings (Table SI4).

### 2.3 Activity Against Planktonic Cells

**FIGURE 2:**
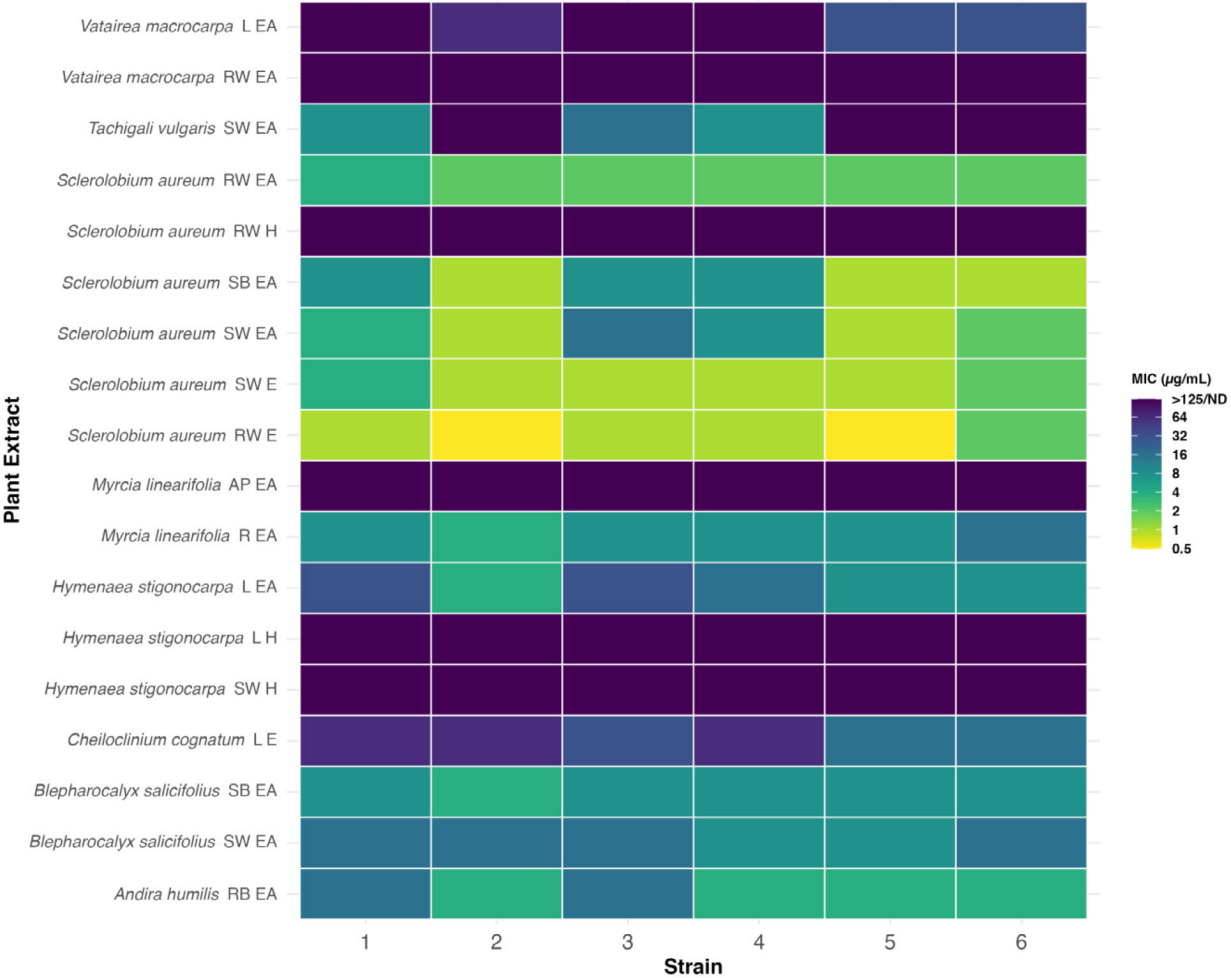
Minimum inhibitory concentrations (MICs) of plant extracts against *Candida* spp. Heatmap shows the MIC values (µg mL^−1^) of the tested plant extracts against clinical and reference strains of *Candida* species. Extracts are indicated on the *Y*‐axis with abbreviated genus names in italics, followed by the plant part and solvent (RB: root bark; SW, stem wood; SB, stem bark; L, leaves; AP, aerial parts; RW, root wood; H, hexane; EA, ethyl acetate; E, ethanol). The *X*‐axis shows the tested strains: 1: *C. albicans* (ATCC 28367); 2: *C. albicans* (ATCC 10231); 3: *C. albicans* (S1 clinical isolate); 4: *C. albicans* (S3 clinical isolate); 5: *C. tropicalis* (LMGO 49); 6: *C. krusei* (LMGO 174). MIC values are color‐coded, with darker shades indicating higher concentrations.

The 18 extracts selected from the initial screening against *C. albicans* biofilm were further evaluated against planktonic cells, with 13 classified as active (MIC ≤ 125 µg mL^−1^), following the threshold established by Albernaz et al. [20]. These 13 extracts inhibited the growth of at least one of the evaluated *Candida* species (Figure 2; Table SI4). Studying planktonic cells is relevant as antifungal agents mainly act on free‐living cells during biofilm disruption [18, 36]. In MIC assays, inhibition of planktonic cell growth reflects an extract's ability to inhibit proliferation in suspension. In developing biofilm assays, inhibition reflects interference with adhesion and early matrix formation, while in mature biofilm assays it indicates disruption of established structures and reduced sessile cells. Together, these assays reveal complementary antifungal effects [7].

*Sclerolobium aureum* (stem wood) ethanol extract exhibited remarkable activity (MIC 0.49 µg mL^−1^) against *C. albicans* ATCC 10231 and *C. tropicalis* LMGO49 (Figure 2; Table SI5). The root wood ethyl acetate extract from the same species also showed strong activity (MIC < 2 µg mL^−1^) against the S1 and S3 clinical isolates. Given the limited phytochemical data available for *S. aureum*, these results underscore its potential as an antifungal candidate, consistently with previous studies reporting its activity against yeast and dermatophytes [15].

Among the other Cerrado species, the *A. humilis* root bark ethyl acetate extract demonstrated activity with MIC values of 15.6 µg mL^−1^ against ATCC 28367 and clinical isolate S1, while showing stronger inhibition (MIC of 3.9 µg mL^−1^) against the other strains tested. *Andira humilis*, commonly known as angelim‐do‐campo, was reported to contain isoflavones and flavonols in its root extracts which exhibit antifungal properties [37]. The H. stigonocarpa leaves ethyl acetate extract also displayed strain‐dependent effects, with MICs of 31.2 and 15.6 µg mL^−1^ against S1 against S3, respectively, and broader activity against nonalbicans *Candida* species (MIC 7.8 µg mL^−1^).

In contrast, some extracts only exhibited moderate inhibition, such as *Vateria macrocarpa* leaves ethyl acetate extract which presented MIC values ranging from 125 to 31.2 µg mL^−1^ (Table SI5). These results highlight strain‐specific antifungal activity (e.g. *A. humilis*, *H. stigonocarpa*), and broad‐spectrum activity (*S. aureum*), emphasizing the importance of evaluating multiple *Candida* strains and species in antifungal screening.

In addition, other Cerrado species also demonstrated relevant antifungal potential. The *C. cognatum* leaves ethanolic extract was particularly effective against non‐*albicans Candida*, with MIC 15.6 µg mL^−1^ against *C. tropicalis* and *C. krusei*, while the same extract showed MIC values of 31.2 µg mL^−1^ (*C. albicans* S1) and 62.5 µg mL^−1^ (*C. albicans* S3). Ethyl acetate extracts from Myrtaceae species, namely the *B. salicifolius* (stem bark and stem wood) and *M. linealifolia* (root extract), also displayed broad activity across all evaluated strains and were more notably effective against clinical *C. albicans* isolates (MIC 7.8 µg mL^−1^) (Table SI5). The low number of studies reporting the antifungal activity of these crude extracts [15, 21, 37, 38], together with our present findings, reinforce the importance of further phytochemical and biological investigations on Cerrado plants.

### 2.4 Inhibition of Yeast‐to‐hyphae Morphological Transition

**FIGURE 3:**
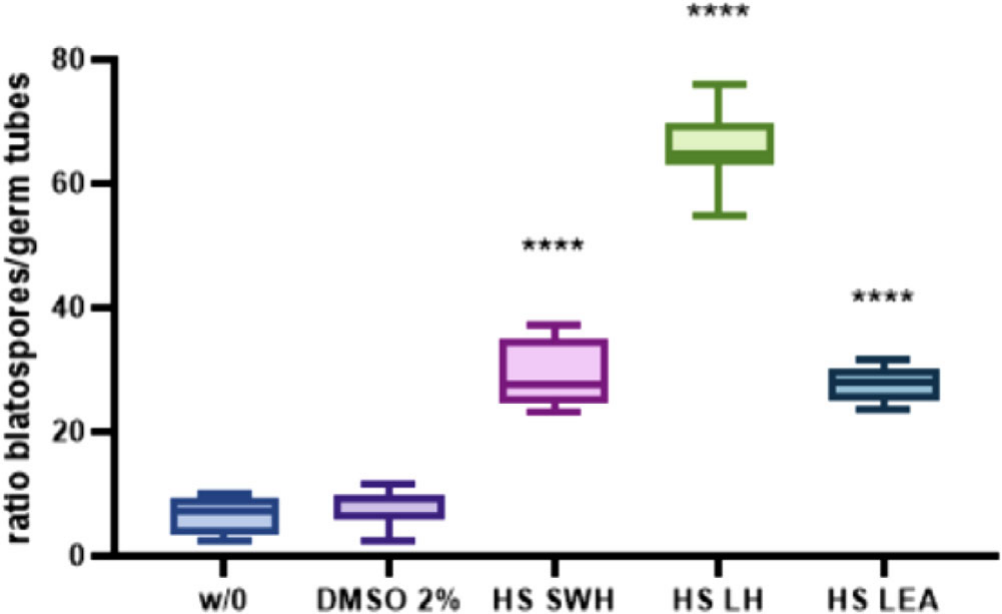
Estimation of the filamentation of *C. albicans* cultured in RMPI with 10% SVF liquid medium treated with dimethyl sulfoxide (DMSO) or extract (200 µg mL^−1^), enumeration by light microscopy. Yeast hyphae form proportion data represent the mean (± standard deviation) of 9 independent experiments. **p < 0.0001. W/0: no treatment; DMSO, dimethyl sulfoxide (vehicle control); HS, *Hymenaea stigonocarpa*; SWH, stem wood hexane, LH, leaf hexane, and LEA, leaf ethyl acetate extracts.

*C. albicans* is a polymorphic fungus capable of undergoing a reversible morphological transition between blastospore and filamentous forms. The ability of *C. albicans* to form hyphae plays a key role in the pathogenesis of candidiasis, as hyphae is crucial for tissue invasion through adherence to host epithelial and endothelial cells [39]. We evaluated the inhibitory effect of the 18 selected extracts under hyphae‐inducing conditions in the S1 strain. The test was performed using RPMI medium supplemented with 10% serum. After incubation at 37°C for 3 h, a statistically significant (*p* < 0.01) inhibition of *C. albicans* morphological transition to the filamentous form was observed in samples treated with H. stigonocarpa extracts, compared to the negative control. The results presented in Figure 3 illustrate the modulation of C. albicans morphogenetic conversion under the influence of the extracts. The species contains diterpene‐class compounds that have been shown to inhibit the yeast‐to‐hyphae transition [40].

### 2.5 Spectral Similarity Networking Evaluation

The active extracts were analyzed by UPLC‐MS/MS (Figure SI1) to identify the ions present. This process improved data visualization by generating a spectral similarity network with clusters corresponding to different compound classes. Clusters are groups of nodes (plotted here as pie charts representing compound percentages in each of the 18 extracts), and the connecting lines constitute edges. In this study, the total molecular networking (MN) (Figure SI2), consists of 29 clusters and 256 selfloops (Task ID 60abc500c0c140dfbda09ba2e227da59). It was possible to annotate 44 compounds by comparing their mass spectra with the GNPS library. An additional 16 compounds were annotated using *in silico* tools [41]. The annotated compounds, 60 in total, are listed in Table 2 (Tabel SI6). The use of cheminformatics tools in natural products research extends beyond compound annotation; it also helps highlight the challenges of classical methods such as separation, isolation, and characterization.

**TABLE 2:** Molecular networking and in silico annotation of compounds in bioactive Cerrado extracts.

Compound	Molecular formula	Experimental *m*/*z*	Rt (min)	Error (ppm)	Extract
**Flavonoids**					
epicatechin	C_15_H_14_O_6_	291.0872	08.63	1.37	ML R EA
catechin	C_15_H_14_O_6_	291.0876	10.69	2.40	SA RW E
epicatechin‐3‐gallate	C_22_H_18_O_10_	443.0989	11.97	2.48	SA SW EA
calycosin	C_16_H_12_O_5_	285.0759	14.21	1.40	VM L EA AH RB EA
formononetin	C_16_H_12_O_4_	269.0811	16.19	1.11	VM L EA VM RW EA
7,4‐methoxy‐3‐hydroxyflavone	C_17_H_14_O_5_	299.0915	16.34	1.34	AH RB EA
5,3'‐dihydroxy‐3,8,4',5'‐tetramethoxy‐6,7‐methylenedioxyflavone	C_20_H_18_O_10_	419.0982	16.58	1.20	ML R EA ML AP EA
3,5‐dihydroxy‐6,7,8‐trimethoxy‐3',4'‐methylenedioxyflavone	C_19_H_16_O_9_	389.0879	16.73	2.05	ML R EA ML AP EA
prunetin	C_16_H_12_O_5_	285.0771	17.69	2.80	VM L EA
biochanin A	C_16_H_12_O_5_	285.0764	17.72	0.35	AH RB EA
tricin	C_17_H_14_O_7_	331.0817	15.33	0.30	HS L EA
chrysoeriol	C_16_H_12_O_6_	301.0702	15.48	3.32	VM L EA
isokaempferide	C_16_H_12_O_6_	301.0721	15.50	2.99	AH RB EA
erythrinin C	C_20_H_18_O_6_	355.1179	13.26	0.56	VM L EA
pseudobaptigenin	C_16_H_10_O_5_	283.0606	16.04	0	AH RB EA
medicarpin	C_16_H_14_O_4_	271.0960	17.11	3.69	AH RB EA
pseudobaptigenin methyl ether	_C17_H_12_O_5_	297.0763	18.33	0.68	AH RB EA
lupiwighteone	C_20_H_18_O_5_	339.1218	18.61	4.13	VM L EA
procyanidin B2	C_30_H_26_O_12_	579.1520	09.85	2.93	SA SB EA
luteolin	C_15_H_10_O_6_	287.0551	14.18	1.74	HS L EA
**Glycosylated flavonoids**					
myricetin 3‐galactoside	C_21_H_20_O_13_	481.0991	9.55	1.87	ML AP EA
myricetin 3‐xyloside	C_20_H_18_O_12_	451.0874	09.93	0.66	ML AP EA
myricetin 3‐rhamnoside	C_21_H_20_O_12_	465.1037	10.01	0.86	ML AP EA
lepidoside	C_26_H_28_O_14_	565.1557	10.62	0	ML AP EA
quercetin 3‐rhamnoside	C_21_H_20_O_11_	449.1088	10.77	0.44	ML AP EA
myricetin 3‐(2”‐acetylrhamnoside)	C_23_H_22_O_13_	507.1150	11.07	0.197	ML AP EA
rutin	C_27_H_30_O_16_	611.1625	11.5	2.12	VM L EA
quercetin 3‐glucoside	C_21_H_20_O_12_	465.1024	11.81	1.94	VM L EA
narcissin	C_28_H_32_O_16_	625.1775	11.89	0.96	HS L EA
nicotiflorin	C_27_H_30_O_15_	595.1660	11.96	0.50	VM L EA
quercetin 3‐O‐(6''‐acetyl‐glucoside)	C_23_H_22_O_13_	507.1133	12.50	1.18	VM L EA
engeletin	C_21_H_22_O_10_	435.1299	12.52	1.84	AH RB EA
afzelin	C_21_H_20_O_10_	433.1144	12.88	2.08	HS L EA
vitexin	C_21_H_20_O_10_	433.1134	10.01	0.23	CC L EA
**Terpenes**					
loliolide	C_11_H_16_O_3_	197.1176	11.23	1.01	ML AP EA CC L E
3‐hydroxyadamantane‐1‐carboxylic acid	C_11_H_16_O_3_	197.1175	12.88	1.5	VM L EA
castanogenin	C_30_H_46_O_6_	503.3361	15.28	2.18	ML AP EA
diospyric acid B	C_30_H4_6_O_6_	503.3387	15.35	2.78	ML R EA
madecassic acid	C_30_H_48_O_6_	469.3306	15.58	3.69	BS SB EA
arjugenin	C_30_H_48_O_6_	487.3425	15.66	0.41	BS SW EA ML R EA
ajunic acid	C_30_H_48_O_5_	489.3567	17.57	2.66	BS SB EA
nivenolide	C_20_H_28_O_4_	333.2036	17.90	9	HS L H
kauradienoic acid	C_18_H_30_O_2_	301.2151	18.66	4.64	HS L H
15‐hydroxy‐kauradienoic acid	C_20_H_28_O_3_	317.2105	19.58	4.7	HS L H
sumaresinolic acid	C_30_H_48_O_4_	455.3529	20.09	0.88	BS SW EA ML REA
ent‐15‐oxo‐16‐kauren‐19‐oic acid	C_20_H_28_O_3_	317.2102	20.11	3.78	HS L H
**Stilbenes**					
resveratrol	C_14_H_12_O_3_	229.0865	10.16	0	ML R EA
5,4'‐dihydroxy‐3,4,3'‐trimethoxybibenzyl	C_17_H_20_O_5_	305.1381	13.29	3.28	TV SW EA
**Xanthones**					
mangiferin	C_19_H_18_O_11_	423.0928	08.78	0.2	CC L EA BS SB EA
3‐O‐methyl mangiferin	C_20_H_20_O_11_	437.1091	09.17	1.6	CC L EA
isomangiferin	C_19_H_18_O_11_	423.0927	10.08	0	CC L EA
**Naphthoquinone**					
dianellidin	C_13_H_12_O_3_	217.0857	12.50		VM RW EA
**Lipids**					
glyceryl palmitate	C_19_H_38_O_4_	331.2835	19.83	3.92	SA RW EA
**Others**					
homovanillyl alcohol 4‐O‐glucoside	C_15_H_22_O_8_	348.1657	08.33	0.86	ML R EA
3,4,5‐trihydroxybenzoic acid	C_7_H_6_O_5_	171.0298	06.95	4.09	ML R EA
ethyl 3,4,5‐trihydroxybenzoate	C_9_H_10_O_5_	199.0609	11.84	1.50	SA RW EA
3,3’,4‐ tri‐O‐methylflavellagic acid	C_17_H_12_O_9_	361.0555	11.92	1.38	ML R EA
ellagic acid	C_14_H_6_O_8_	303.0137	10.31	1.32	BS SW EA
chlorogenic acid	C_16_H_18_O_9_	355.1035	08.55	1.69	ML AP EA

**Abbreviations**: AH, *A. humilis*; AP, aerial parts; BS, *B. salicifolius*; CC, *C. cognatum*; E, ethanol; EA, ethyl acetate; H, hexane; HS, *H. stigonocarpa*; L, leaves; ML, *M. linearifolia*; R, root; RB, root bark; Rt, retention time; RW, root wood; SA, *S. aureum*; SB, stem bark; SW, stem wood; TV, *T. vulgaris;* VM, *V. macrocarpa*.

**FIGURE 4:**
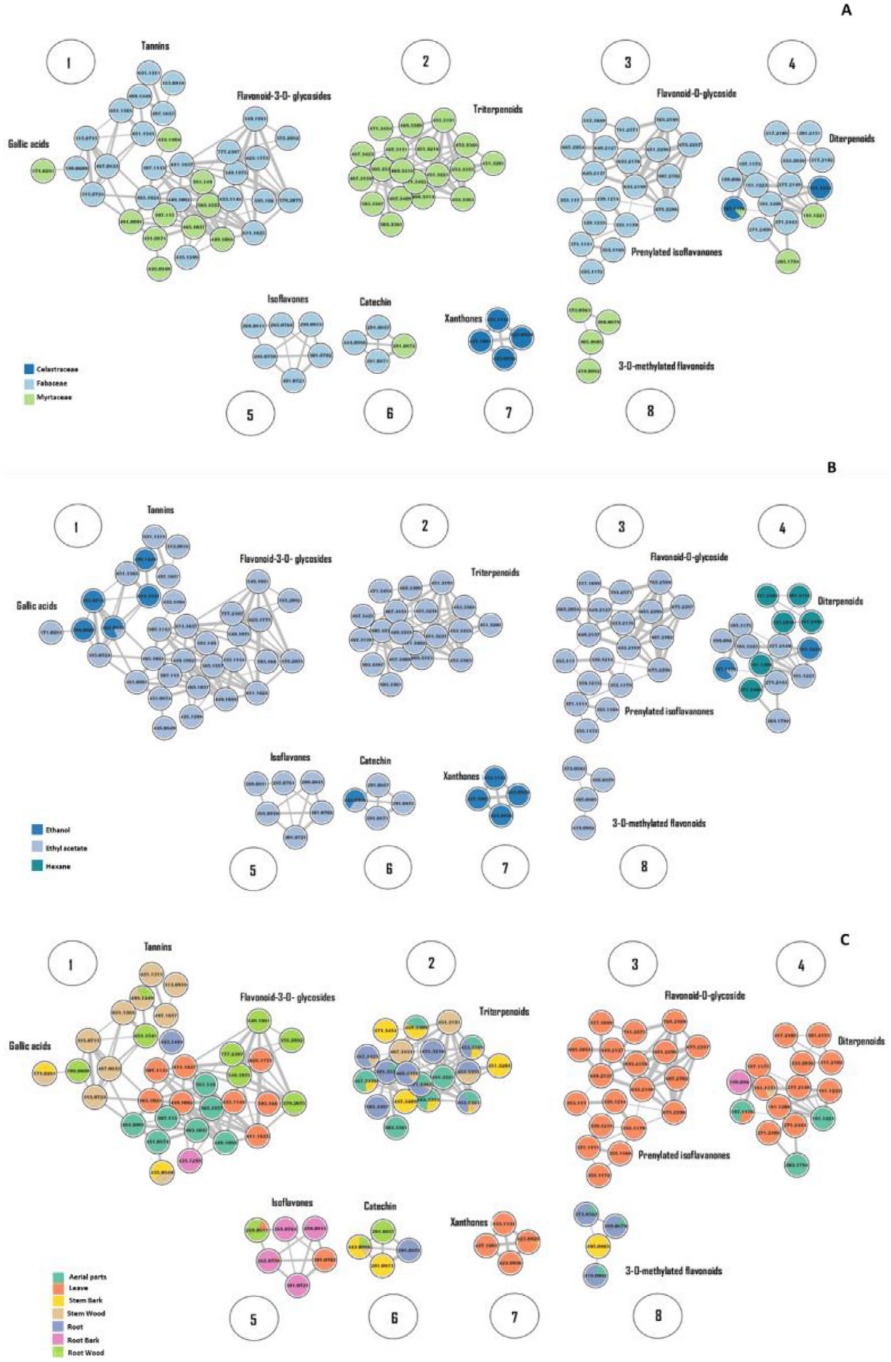
Clusters obtained by feature‐based molecular networking (FBMN) highlight the main classes of compounds annotated in the active extracts. (A) Nodes are separated according to the plant family where they were identified (Fabaceae, Myrtaceae, or Celastraceae). (B) Nodes are separated by solvent polarity: hexane, ethyl acetate, or ethanol. (C) Nodes are separated by the plant organs from which they were annotated: aerial parts, leaves, root (bark or wood), and stem (bark or wood).

Figure 4 presents eight molecular clusters obtained by feature‐based molecular networking (FBMN), using the parameters defined in this study, visualized under 3 perspectives: (A) plant families (Fabaceae, Myrtaceae, and Celastraceae); (B) extraction solvents (hexane, ethyl acetate, and ethanol), and (C) plant organs (root, stem, leaves/aerial parts). In Figure 4A, glycosylated flavonoids (cluster 1), prenylated flavonoids (cluster 2), isoflavones (cluster 3), catechins (cluster 4), and diterpenoids (cluster 5) predominated in Fabaceae, while triterpenoids (cluster 6) and O‐methylated flavonoids (cluster 7) were exclusive to Myrtaceae, while xanthones (cluster 8) were exclusive to Celastraceae.

As shown in Figure 4B, solvent polarity influenced chemical profiles: diterpenoids dominated in hexane; ethanol extracts contained tannins, diterpenoids, xanthones, and catechins, while ethyl acetate revealed the broadest diversity. These findings are consistent with previous reports in the literature [42, 43]. Finally, Figure 4C highlights organ‐specific distribution, with xanthones, prenylated isoflavones and diterpenes enriched in leaves; nonprenylated isoflavones in roots; triterpenes in both aerial parts and roots; tannins in stems/roots, and glycosylated flavonoids across all organs.

The compound classes annotated in this study are consistent with previously reported phytochemistry for the evaluated genera. Prenylated isoflavones have been described in *Vatairea* spp. [15, 44, 45]; biochanin A is a chemotaxonomic marker of *A. humilis* [37]; flavonoid glucosides and terpenoids are major classes in *Myrcia* spp. [46], and mangiferin is well documented in Celastraceae [47, 48].

### 2.6 Chemical Variation Among Plant Species and Its Relationship With Antifungal Activities

**FIGURE 5:**
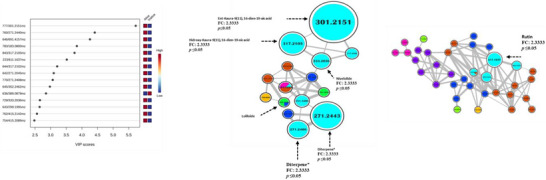
Analysis of variable importance in projection (VIP) scores and molecular networks obtained from MetaboAnalyst.ca and the GNPS online platform. (A) VIP scores generated by PLS‐DA analysis, highlighting the most discriminant features based on their relative abundance. (B) Molecular network of the mono‐ and diterpene cluster. (C) Molecular network of the flavonoid cluster.

The data were analyzed using the MetaboAnalyst platform (www.metaboanalyst.ca). Partial Least Squares Discriminant Analysis (PLS‐DA) combined with Variable Importance in Projection (VIP) scores identified ions that best discriminated extracts inhibiting/not inhibiting hyphal formation. A t‐test and Fold Change (FC) analysis (threshold > 2.0 or < –2.0) further supported their statistical significance (*p* ≤ 0.05), suggesting these ions as putative biomarkers rather than confirmed bioactive compounds (Figure 5A). Among them, several diterpenoids were consistently annotated in *H. stigonocarpa* extracts, including kauradienoic acid (*m*/*z* 301.2151), 15α‐hydroxy‐kauradienoic acid (*m*/*z* 317.2105), nivenolide (*m*/*z* 333.2036), and 2 additional diterpenoid ions (*m*/*z* 271.2443 and 271.2408), together with a glycosylated flavonoid (*m*/*z* 611.1637) (Figure 5b,C). Previous phytochemical reports confirmed the presence of diterpenes and flavones in *H. stigonocarpa* [40, 49, 50] compounds known to interfere with *C. albicans* virulence and morphogenesis [51, 52].

**FIGURE 6:**
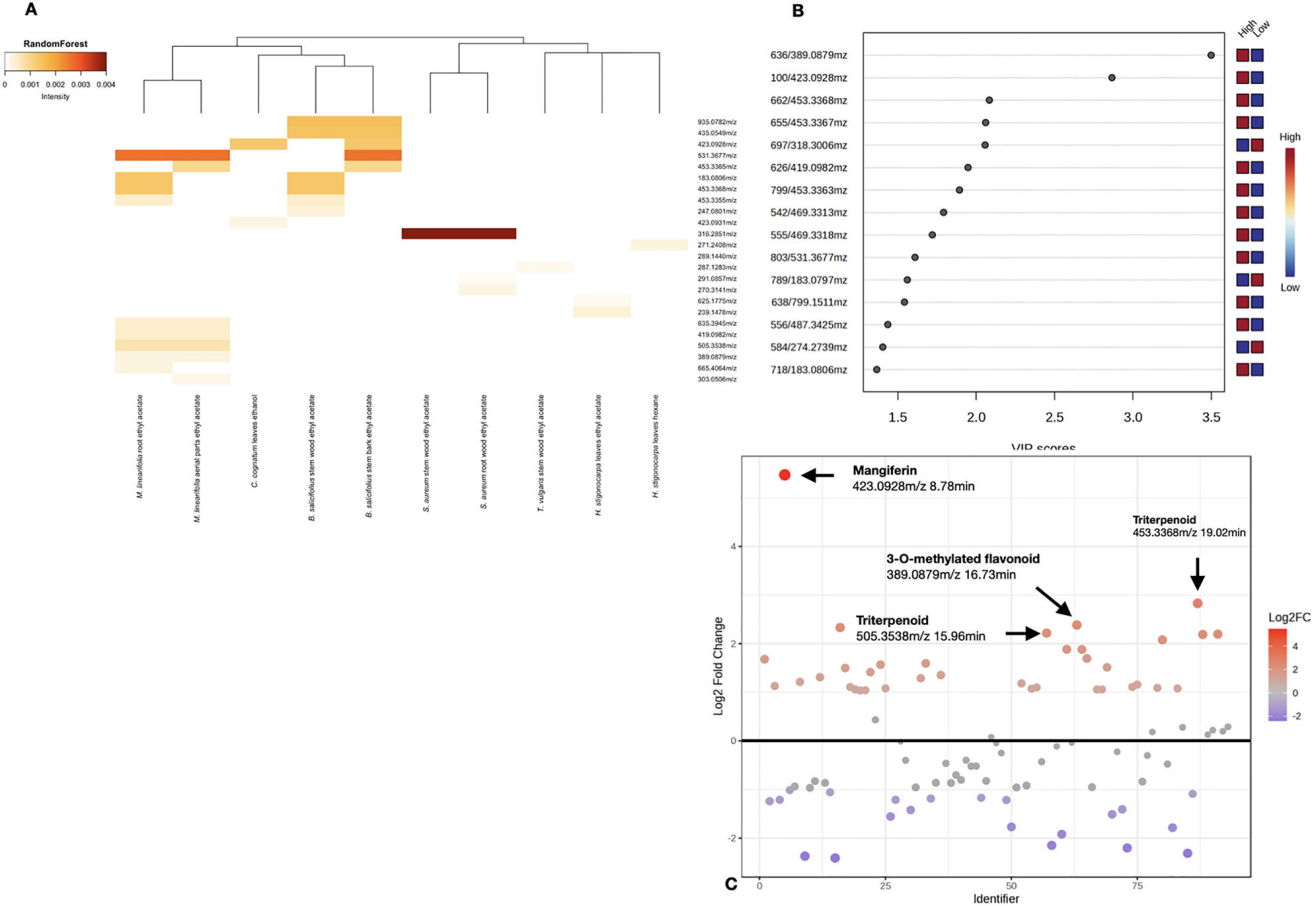
(A) Hierarchical clustering based on ion intensities in *Cerrado* plant extracts, showing differential metabolite abundance associated with antibiofilm activity against clinical *C. albicans* strains S1 and S3. (B) Variable Importance in Projection (VIP) scores from PLS‐DA highlighting ions most strongly contributing to group separation. **(C)** Log_2_ Fold Change analysis identifying key ions with significant variation (|log_2_FC| > 1), annotated as triterpenoids, xanthones, and methylated flavonoids.

No statistically significant ions associated with biofilm inhibition in clinical isolates were detected (*p* ≤ 0.05), likely due to chemical heterogeneity and marginal differences in bioactivity scores. However, Random Forest analysis (FBMN STATS GUIde) suggested ions potentially linked to this phenotype (Figure 6A–C). Heatmap visualization (Figure 6A) revealed ions with species‐specific distributions, also identified in Fold Change (Figure 6B) and VIP‐score plots (Figure 6C). These included mangiferin (*m*/*z* 423.0928) from the *C. cognatum* leaf ethanolic extract, and a 3‐O‐methylated flavonoid (*m*/*z* 389.0879) from the *M. linealifolia* root ethyl acetate extract. Mangiferin has documented antifungal activity against azole‐resistant *Candida* species and synergism with amphotericin B [53], while methylated flavonoids are reported to display enhanced absorption and activity compared to their nonmethylated analogues [54]. Other nodes annotated by Sirius (*m*/*z* 419.0982, *m*/*z* 373.0563, *m*/*z* 465.1037, and *m*/*z* 611.1637) were consistent with 3‐O‐methylated or 3‐O‐glycosylated flavonoids, classes known to disrupt fungal membranes or deregulate homeostasis [55]. Nevertheless, these annotations should be interpreted as chemical markers correlated with activity rather than direct proof of causality. SPE‐Diol fraction and bioassay‐guided isolation will be required to verify whether these ions represent the compounds responsible for the antifungal effects of the extracts.

*Hymenaea stigonocarpa* synthesizes a diverse array of secondary metabolites, including diterpenes, triterpenes, flavonoids, and stilbenes, which have been associated with antimicrobial and anti‐inflammatory properties [28, 50, 56]. *Cheiloclinium cognatum* predominantly accumulates pentacyclic triterpenes and alkaloids, compounds known for their antifungal and cytotoxic effects [23, 30]. Members of the Myrtaceae family, such as *B. salicifolius* and *M. linearifolia*, are chemically characterized by high concentrations of triterpenes, flavonoids, and essential oils rich in monoterpenes and sesquiterpenes, which contribute to their notable antioxidant, antifungal, and anti‐inflammatory activities [31, 46].

### 2.7 In Silico Activity

**FIGURE 7:**
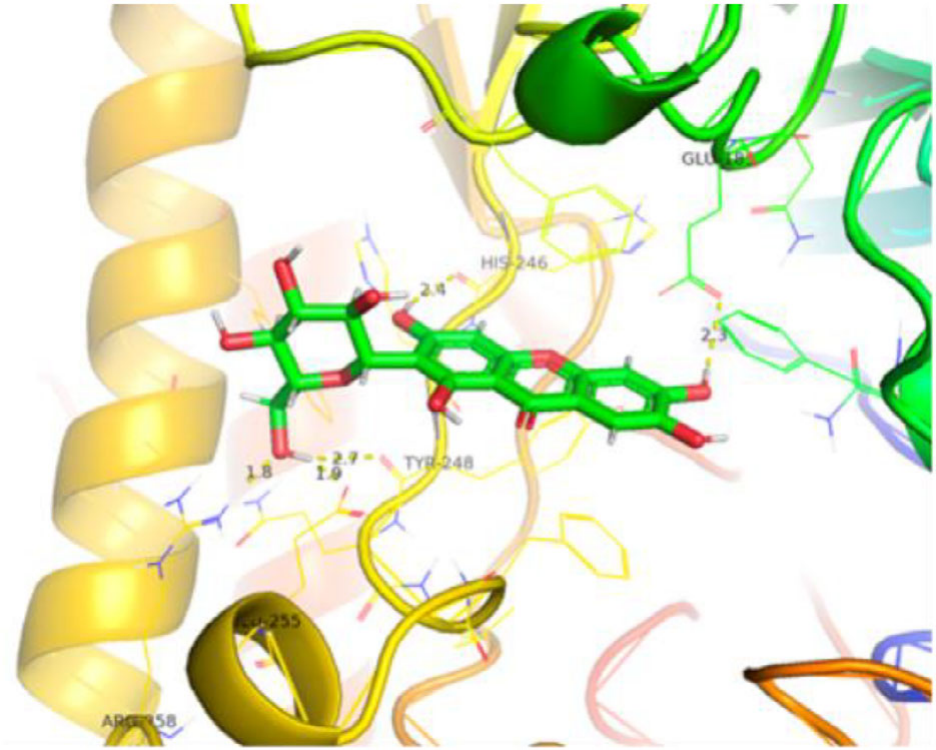
Interaction of enzyme residues with mangiferin. The binding interactions occur with Arg258 (1.8 Å), Glu255 (1.9 Å), Gly185 (2.3 Å), His246 (2.4 Å), and Tyr248 (2.7 Å).

Binding interaction analyses revealed that Arg258 in the β1,3glucanase enzyme forms a hydrogen bond with mangiferin at approximately 1.8 Å, indicating a strong and potentially crucial interaction for complex stability. Similarly, Glu255 engages at around 1.9 Å, reinforcing mangiferin's pronounced affinity for the enzyme. Additional hydrogen bonds were observed with Gly185, His246, and Tyr248 at distances of 2.3, 2.4, and 2.7 Å, respectively, underscoring their roles in anchoring and molecular recognition. Consistent with these findings, mangiferin exhibited a binding energy of −8.8 kcal mol^−1^, highlighting its robust interaction with key residues. In contrast (4S,5S,10S)‐5,9‐dimethyl‐14‐methylidene‐15‐oxotetracyclo [11.2.1.01,10.04,9] hexadecane‐5‐carboxylic acid forms 3 hydrogen bonds, with Arg258 establishing two (2.4 and 1.1 Å) and Trp270 contributing a third (1.1 Å). This ligand demonstrated a binding energy of −7.1 kcal mol^−1^, indicating a noteworthy, however distinct interaction profile relative to mangiferin. For comparison, grandiflorenic acid yielded a single hydrogen bond with Trp270 (0.8 Å) and a binding energy of −6.9 kcal mol^−1^ (Figure 7). Our docking studies indicate that mangiferin binds to *Candida albicans* exoβ1,3glucanase with a binding energy of −8.8 kcal mol^−1^, comparable to the −8.8 kcal mol^−1^ observed for compound 3b, 5(4chlorobenzylidene)‐3((Z)‐((E)‐3phenylallylidene)amino)imidazolidine‐2,4dione, docked with the *C. albicans* dihydrofolate reductase (PDB ID: 1AI9). In the antifungal assays, compound 3b exhibited potent activity against *C. albicans* (MIC 0.25 µg mL^−1^). These results suggest that both ligands interact with their target proteins with similar affinity, supporting their potential as leads in antimicrobial drug discovery against *C. albicans*.

Although (4S,5S,10S)‐5,9‐dimethyl‐14‐methylidene‐15‐oxotetracyclo[11.2.1.01,10.04,9]hexadecane‐5‐carboxylic acid and grandiflorenic acid also engage the enzyme, they exhibit fewer hydrogen bonds and some longer bond distances, resulting in distinctly weaker net interactions [57].

For kauren and mangiferin, notable binding to enzymes of medical relevance was identified. Kauren, targeting 11β‐hydroxysteroid dehydrogenase type 1 (11β‐HSD1), showed a binding energy of −9.1 kcal mol^−1^, forming hydrogen bonds with key residues Lys20 (2.3 Å) and Arg42 (1.7 and 2.6 Å). In parallel, mangiferin, when docked with aldose reductase, achieved a binding energy of −8.1 kcal mol^−1^, with significant interactions observed with Trp220 (1.7 and 2.3 Å), Phe312 (2.8 Å), Asp225 (1.7 Å), Arg218 (2.5 and 2.4 Å), and Lys222 (2.8 Å). The development of inhibitors for these enzymes is of considerable therapeutic interest.

To expand the in silico analysis, ADMET, and physicochemical properties of mangiferin were predicted using the SwissADME platform (Table 3). Mangiferin showed characteristics compatible with interaction with soluble enzymatic targets, including high polarity (TPSA = 201.28 Å^2^), high aqueous solubility, and low lipophilicity [58]. These features may favor interactions with solvent‐accessible proteins rather than membrane‐embedded targets. Such findings are consistent with previous reports describing mangiferin as an inhibitor of soluble carbohydrate‐metabolizing enzymes and support its potential interaction with fungal enzymes involved in cell wall remodeling, including exo‐β‐1,3‐glucanase.

**TABLE 3:** Physicochemical and ADMET properties of mangiferin predicted using the SwissADME web server, including solubility, lipophilicity (LogP), topological polar surface area (TPSA), cytochrome P450 inhibition, P‐glycoprotein substrate prediction, and drug‐likeness parameters.

	Mangeferin	Kauren	Hexadecane‐5‐carboxylic acid
**Water Solubility**			
Log S (ESOL):	−2.44	−4.57	−4.51
Solubility (ESOL):	1.54 mg/mL	8.16e−03 mg/mL	9.68e−03 mg/mL
Log S (Ali):	−3.39	−5.16	−5.27
Solubility (Ali):	0.17 mg/mL	2.08e−03 mg/mL	1.70e−03 mg/mL
Log S (SILICOS‐IT):	−0.97	−4.09	−4.00
Solubility (SILICOS‐IT):	45.2 mg/mL	2.41e−02 mg/mL	3.20e−02 mg/mL
Class:	Soluble	Moderately soluble	Soluble
**Lipophilicity**			
iLOGP:	0.89	2.84	2.52
XLOGP3:	−0.37	4.65	4.41
WLOGP:	−1.04	4.96	4.22
MLOGP:	−2.66	4.54	3.66
SILICOS‐IT LogP:	−0.68	4.32	4.02
Consensus LogP:	−0.77	4.26	3.77
**Cytochrome P450 inhibition**			
CYP1A2 inhibitor:	No	No	No
CYP2C19 inhibitor:	No	Yes	No
CYP2C9 inhibitor:	No	Yes	Yes
CYP2D6 inhibitor:	No	No	No
CYP3A4 inhibitor:	No	No	No
**P‐glycoprotein (P‐gp)**			
P‐gp substrate:	No	No	Yes
**Structural stability**			
Number of rotatable bonds:	2	1	1
**Synthetic accessibility**			
Synthetic accessibility score:	4.76	5.75	5.05
**TPSA (Topological Polar Surface Area)**	201.28 Å^2^	37.30 Å^2^	54.37 Å^2^

## 3 Conclusions

This study evaluated 108 plant extracts from the Cerrado biome and demonstrated the significant antifungal and anti‐biofilm potential of 18 extracts from native and endemic species against *Candida* species. Notably, the Fabaceae family showed pronounced biofilm inhibition, particularly the ethyl acetate extract of *T. vulgaris* stem wood, while *S. aureum* extracts exhibited strong activity against both planktonic and biofilm forms. Given its broad‐spectrum efficacy against *C. albicans* and non‐*albicans* species, *S. aureum* stands out as a promising candidate for further investigation. Chemical profiling by UPLC‐MS/MS identified key bioactive compounds, such as phenolics, flavonoids, and terpenes, reinforcing their potential as bioactive molecules of pharmacological interest. *In silico* docking analyses supported these findings by revealing favorable binding affinities of these compounds to fungal target proteins. Although some resistant clinical strains exhibited reduced susceptibility, the overall findings underscore the relevance of natural products in the search for novel therapeutic agents. Furthermore, the promising affinities of kaurenoic acid and mangiferin suggest their potential applications beyond antifungal therapy, including in the treatment of metabolic and diabetic disorders. In summary, this study highlights the ecological and pharmacological value of the Cerrado biome and supports continued bioprospecting efforts for innovative treatments against fungal infections, especially in the context of rising antifungal resistance.

## 4 Experimental Section

### 4.1 Plants Extracts

Plant material was collected in the Cerrado biome near Brasília, DF, Brazil by the botanist Professor José Elias de Paula (*in memoriam*), and voucher specimens were deposited in the University of Brasília (UB/UnB) Herbarium. Plant organs were separated, dried, then pulverized and extracted by maceration with solvents of different polarities. Extract solutions were concentrated with a rotary evaporator at 35°C to yield crude extracts, stored at −20°C. The Laboratory of Pharmacognosy of the University of Brasília holds a license granted by CGEN/IBAMA No. 06/2012–Process No. 02000.002272/2006‐73 (Table 4).

**TABLE 4:** A total of 108 plant extracts from 22 species across 12 families (in this table) were evaluated against *C. albicans* ATCC 28367 biofilms.

Family	V.N.	Plant species	Occurrence	Plant parts (solvent)
Bignoniaceae	UB3799	*Zeyheria montana* Mart.	endemic	RB (EA); RW (H); S (E/ EA/H); L (H)
Celastraceae	UB3805	*Cheiloclinium cognatum* (Miers) A. C. SM.	native	SW (EA/H/E); SB (H/W); L (EA/H/E)
	UB3747	*Plenckia populnea* Reissek	native	SB (EA/E); L (EA/ E); SW (H/EA)
Dilleniaceae	UB3773	*Davilla elliptica* A. ST.‐Hill	native	L (EA/H/E); SW (E); SB (H)
Fabaceae	UB3764	*Andira humilis* Mart. ex Benth	endemic	RB (E); L (E); RW (H); SW (EA/E); SB (E); RB (EA/H)
	UB3763	*Andira vermifuga* Mart. ex Benth	native	L (H)
	UB3800	*Chamaecrista desvauxii* (Collad.) Killip	native	AP (EA/E/H)
	UB1171353	*Hymenaea stigonocarpa* Mart. ex Hayne	endemic	L (H/EA); SB (H)
	UB3818	*Sclerolobium aureum* (Tul.) Benth.	native	RW (EA/H/E); SW (EA/H/E); SB (EA); RB (EA); L (EA)
	UB1061912	*Tachigali vulgaris* L.G.Silva & H.C.Lima	endemic	L (EA/ H/E); SW (EA/H); SB (EA/H)
	UB3815	*Vatairea macrocarpa* (Benth.) Ducke	native	L (EA); RB (EA); RW (EA/H); SB (EA); SW (E/H)
Myrtaceae	UB3798	*Blepharocalyx salicifolius* (Kunth) O.Berg	native	L (EA/H/E/W); SW (EA); SB (EA/H); RB (W)
	UB3803	*Eugenia dysenterica* DC.	native	L (Hy)
	UB3817	*Myrcia linearifolia* Cambess.	endemic	R (AE); AP (EA)
Nyctaginaceae	UB1537	*Guapira noxia* (Netto) Lundell	endemic	SW (EA); RW (EA); RB (EA)
	UB3821	*Neea theifera* Oerst.	native	RB (EA); RW (EA/H/E); SW (EA/H); L (EA)
Ochnaceae	UB3713	*Ouratea floribunda* (A.St.‐Hil.) Engl.	endemic	F (H); L (D)
Opiliaceae	UB3797	*Agonandra brasiliensis* Miers ex Benth. & Hook.f.	native	L (EA/H/E)
Sapotaceae	UB3733	*Chrysophyllum soboliferum* Rizzini	native	L (E/H)
Symplocaceae	UB3331	*Symplocos rhamnifolia* A.DC.	endemic	SB (EA); SW (EA); RB (EA); RW (EA)
Verbenaceae	UB3796	*Lippia rotundifolia* Cham.	endemic	R (H); SB (EA); SW (EA/H); L (EA/H/E); Fl (EA/H)
Vochysiaceae	UB3777	*Salvertia convallariodora* A.St.‐Hil.	native	L (EA/E); SW (EA/E)

**Abbreviations**: AP, aerial parts; D, dichloromethane; E, ethanol; EA, ethyl acetate; F, fruit; H, hexane; Hy, hydroalcoolique; L, leaves; R, root; RB, root bark; RW, root wood; S, stem; SB, stem bark; SW, stem wood; V.N., Voucher Number; W, water.

### 4.2 Microbial Strains and Culture Conditions

Activity against *C. albicans* reference strains (ATCC 28367 and ATCC 10231) and clinical isolate strains (S1 and S3) obtained from patient catheter samples from the Laboratory of Parasitology and Medical Mycology, University Hospital of Poitiers (France). We also included clinical isolates of *C. tropicalis* (LMGO 49) and *Candida krusei* (LMGO 174) collected from patients treated at the University Hospital of the Federal University of Goiás (UFG). Prior to all assays, yeast cells were subcultured on Sabouraud Dextrose Agar and incubated at 35°C for 24 h.

For the planktonic susceptibility assay, yeast inoculum was adjusted by turbidimetry to a transmittance of 60%–65% at 530 nm. The suspensions were diluted 1:100, followed by a 1:20 dilution in RPMI 1640 medium (Sigma‐Aldrich), yielding a final concentration of 1 × 10^4^ CFU mL^−1^, according to CLSI guidelines [59]. For biofilm formation, *C. albicans* ATCC 10231 and clinical isolate strains (S1 and S3) were grown overnight at 37°C in YNB medium supplemented with glucose under static conditions. Cells were then washed twice with phosphate‐buffered saline (PBS) by centrifugation at 2000 rpm for 10 min and quantified using Fast‐Read 102 counting chambers (Biosigma, Cantarana, Italy) [17].

### 4.3 *Candida albicans* Biofilm

Biofilm formation and inhibition assays were performed following the protocol described by Hamion et al. [17], with minor modifications. Briefly, *C. albicans* biofilms were cultured in 96‐well polystyrene nontreated microtiter plates (Costar, Corning, NY, USA). For biofilms, 200 µL of cultures at 1 × 10^7^ cells mL^−1^ were inoculated in each well. To evaluate activity against biofilm development, the culture medium was removed and fungal cells washed with PBS after incubation at 37°C for 2 h to remove planktonic cells. Evaluation of activity against mature biofilm was performed in the same way, but after 24 h of incubation at the same temperature. The tested sample consisted of the plant extract suspended in dimethyl sulfoxide (DMSO). A 300 µL aliquot of YNB‐Glu liquid medium with 1.25% DMSO (condition control), caspofungin (positive control) or plant extract suspended in DMSO was added to wells, while some wells only containing biofilm were preserved (negative control). Plates were incubated for 48 h at 37°C. After 48 h of treatment, spent medium and planktonic cells were removed by aspiration, and wells were gently washed with PBS. Biofilm structure and integrity were qualitatively assessed using an inverted optical microscope (IX51, Olympus America Inc., Melville, NY, USA). Quantitative biofilm analysis was performed using a metabolic assay based on the reduction of the tetrazolium salt XTT. Briefly, 50 µL of an XTT/menadione solution was added to each well containing 200 µL of PBS. Plates were incubated at 37°C for 3 h in the dark. The reduction of XTT to formazan by metabolically active biofilm cells was quantified spectrophotometrically at 492 nm using a microplate reader (LP400; Sanofi Diagnostics Pasteur). Background absorbance was determined in wells containing only PBS and XTT/menadione and did not exceed 0.05 absorbance units. In this study, plant extracts exhibiting ≥ 50% inhibition of biofilm formation relative to untreated controls were considered active. All extracts were tested against both developing and mature biofilms of *C. albicans* ATCC 10231. However, only the active extracts were selected for further testing against clinical isolates, planktonic, and hyphal cells, and subjected to chemical analysis.

### 4.4 Bioactive Score

The Bioactive Score (BS) was calculated by normalizing inhibition values relative to the most active sample (*Imax*), using the formula:

$$\mathrm{BioactiveScore}=\left(I-SD\right)\times 100/\mathrm{Imax}-\mathrm{SDImax}$$

where *I* is the percentage of inhibition and *SD* the standard deviation of each condition. A BS of 100 was assigned to the most active sample. Extracts with BS ≥ 60 were classified as active, a threshold supported by sensitivity analyses comparing BS ≥ 50 and BS ≥ 70 (Table SI3). To evaluate biological relevance, BS values were further compared with antifungal endpoints such as MIC and hyphal inhibition assays (Table SI4). Scores were integrated into the spectral feature table obtained with MZmine for subsequent annotation and prioritization of metabolites. Full details of the validation procedure are provided in the Supporting Information (SI).

### 4.5 Inhibition of Yeast‐to‐hyphae Morphological Transition

The ability of plant extracts to inhibit the yeast‐to‐hypha morphological transition of *C. albicans* was assessed following the protocol described by Hamion et al. [27]. *Candida albicans* ATCC 10231 and clinical isolate strains (S1 and S3) were grown overnight in YNB–glucose medium at 37°C, harvested by centrifugation, washed with PBS, and resuspended in RPMI 1640 medium supplemented with GlutaMAX (Gibco, Invitrogen) and 10% fetal bovine serum (FBS) to a final concentration of 10^6^ CFU mL^−1^. Cell suspensions were incubated for 3 h at 37°C in the presence of 2% DMSO (vehicle control), crude plant extracts (200, 100, or 50 µg mL^−1^), or 25 mM EDTA (positive control), a known inhibitor of hyphal formation [60]. Following incubation, 10 µL of each culture were loaded onto Fast‐Read 102 counting chambers (Biosigma, Italy). The proportions of yeast and hyphal forms were quantified microscopically by counting cells in six independent fields per replicate.

### 4.6 Minimum Inhibitory Concentration (MIC) for Planktonic Cells

The minimal inhibitory concentration (MIC) was determined using the broth microdilution method in accordance with Clinical and Laboratory Standards Institute (CLSI) guidelines [59]. Assays were conducted in 96‐well microtiter plates using RPMI 1640 medium (Sigma‐Aldrich) buffered to pH 7.0 with MOPS (3‐(N‐morpholino)propanesulfonic acid, Sigma‐Aldrich). Itraconazole and fluconazole (Sigma‐Aldrich) were included as positive control antifungals.

Plant extracts were dissolved in dimethyl sulfoxide (DMSO) to a stock concentration of 25 mg mL^−1^ and subsequently serially diluted (two‐fold) across a range of 1000 to 0.122 µg mL^−1^. Each well received 100 µL of the respective dilution and an equal volume of standardized fungal inoculum, prepared as described above, to reach a final concentration of 1 × 10^4^ CFU mL^−1^. Microplates were incubated at 37°C for 48 h, and MIC values were determined by visual inspection as the lowest concentration that completely inhibited fungal growth. Negative (medium only) and positive (medium + inoculum with antifungal) controls were included on each plate.

### 4.7 Statistical Analysis

The Kruskal–Wallis test followed by Dunn's multiple comparisons post hoc test was performed using GraphPad Prism, version 10 (GraphPad Software, San Diego, CA, USA), to evaluate differences in antibiofilm and anti‐filamentation activities. Data are presented as mean ± standard deviation (SD) from at least three independent experiments. Differences were considered statistically significant at p < 0.05. All in vitro assays, including minimum inhibitory concentration (MIC) determination, biofilm inhibition, and yeast‐to‐hyphae transition, were conducted in triplicate and independently repeated at least three times to ensure reproducibility. Unless otherwise stated, results are expressed as mean ± SD. For planktonic susceptibility assays, MIC values were determined by visual inspection according to CLSI guidelines [59]. As this method provides qualitative endpoints, no statistical analysis was performed for these data. For comparisons between biofilm formation and mature biofilm inhibition for each extract are additionally presented with 95% confidence intervals. Pairwise comparisons were conducted using Welch's unequal variances *t*‐test, with statistical significance set at *p* < 0.05.

### 4.8 Chemical Analysis

#### 4.8.1 HRESIMS Data Acquisition

Extracts active against biofilms were dissolved in methanol (1 mg mL^−1^), filtered through a 0.22 µm membrane (Millipore), and analyzed by UHPLC–MS/MS. All extract samples were analyzed in duplicate. Methanol blank injections were performed throughout the analytical sequence to monitor background signals, contamination, and potential carryover. A 10 µL aliquot was injected into a UHPLC system (Elute UHPLC pump and autosampler, Bruker Daltonics) coupled to an ESI‐qTOF mass spectrometer (Compact QTOF, Bruker Daltonics) and equipped with a diode array detector (DAD; 200–700 nm scan range).

Chromatographic separation was performed on an Intensity Solo 1.8 C18‐2 column (100 × 2.1 mm, 1.8 µm particle size, 100 Å pore size; Bruker Daltonics), protected by a matching precolumn. The column temperature was maintained at 40°C, and the autosampler was set at 20°C. The mobile phase consisted of solvent A (ultrapure water with 0.1% acetic acid) and solvent B (methanol with 0.1% acetic acid), using a linear gradient from 5% to 95% B over 30 min. The system was equilibrated with 5% B for 1 min before and after the run, at a flow rate of 0.5 mL/min.

Mass spectra were acquired in positive ion mode over an *m*/*z* range of 50–1300 using the Auto_MS method. Source parameters included a desolvation gas flow (N_2_) of 9 L min^−1^, a source temperature of 220°C, and a capillary voltage of 4.5 kV. The cone voltage and collision energy were set at 4 and 7 eV, respectively. The collision cell pressure was 1.8 bar, and the transfer time was 5 µs. Data were processed using DataAnalysis v4.4 (Bruker Daltonics).

#### 4.8.2 Data Processing and Spectral Similarity Network Construction

High‐resolution mass spectrometry (HRMS), combined with molecular networking (GNPS), enables efficient dereplication and prioritization of bioactive metabolites [61]. This integrative approach correlates bioactivity with chemical profiles, accelerating discovery of antifungal leads [62]. Extracts exhibiting activity against *Candida* biofilms were analyzed by UPLC–MS/MS in positive ion mode under the conditions described above. Raw ESI‐MS/MS data were converted to the .mzXML format using DataAnalysis.

Data processing was performed using MZmine 4.0.8, including peak detection, chromatogram deconvolution, deisotoping, blank subtraction, and gap filling. Features detected in methanol blank injections were removed during blank subtraction prior to downstream statistical analyses. Parameters were set as follows: mass detection in centroid mode; chromatogram building with a minimum time span of 0.05 min, minimum peak height of 1000, and *m*/*z* tolerance of 5 ppm. The Local Minimum Resolver (LMR) algorithm was applied for chromatogram deconvolution with a chromatogram threshold of 85%, peak duration range of 0.05–2 min, minimum relative height of 0, minimum absolute height of 3000, and minimum peak top/edge ratio of 1. Isotopic peaks were grouped with an *m*/*z* tolerance of 5 ppm and retention time (RT) tolerance of 0.05 min. Peak alignment utilized the Join aligner method with *m*/*z* tolerance of 5 ppm, absolute RT tolerance of 0.05 min, weighted 75% for *m*/*z* and 25% for RT. Gap filling was performed with an intensity tolerance of 20%, *m*/*z* tolerance of 5 ppm, and RT tolerance of 0.05 min. Processed data were exported as *.csv and *.mgf files containing MS1 and MS2 spectra and tables compatible with the MetaboAnalyst web platform.

Molecular networking was conducted on the GNPS platform (GNPS2 – Analysis Hub, accessed November 4, 2024) using the Feature‐Based Molecular Networking (FBMN) workflow. Active compound prediction was performed as described by Nothias et al. [62]. For molecular network construction, data clustering parameters were set as follows: precursor ion tolerance of 0.01 Da; fragment ion mass tolerance of 0.01 Da; minimum cosine score threshold > 0.7; and minimum topK set to 10. The resulting molecular network was visualized using Cytoscape software (v. 3.10.3). In addition to GNPS spectral libraries, molecular annotation of potential bioactive compounds was performed using the in silico tool SIRIUS. Quantitative area values were analyzed in MetaboAnalyst 6.0 (McGill University, Montreal, Canada). Data scaling was performed using Pareto scaling (mean‐centering and division by the square root of the standard deviation for each variable) without further data transformation. This approach was specifically employed to evaluate fold change and quantify the relative magnitude of metabolite abundance alterations between experimental groups. Samples were grouped based on their activity profiles, specifically hyphal inhibition or antibiofilm activity. This analytical workflow was designed for exploratory metabolite profiling and dereplication of bioactive extracts.

### 4.9 In Silico Activity

The exo‐β‐1,3‐glucanase structure (PDB ID: 4M82) was prepared using the pdb2pqr protocol [63] at pH 7.0 to assign appropriate protonation states. Ligands selected for docking included Mangiferin (PubChem CID: 5281647), Kauren‐9(11),16‐dien‐19‐oic acid (Grandiflorenic acid, PubChem CID: 9994991), and (4S,5S,10S)‐5,9‐dimethyl‐14‐methylidene‐15‐oxotetracyclo[11.2.1.0^1^,^10^.0^4^,^9^]hexadecane‐5‐carboxylic acid (PubChem CID: 11969647), all retrieved from the PubChem database. Docking simulations were conducted using the DockThor platform. Protein–ligand interactions, including hydrogen bond formations, were analyzed and visualized with PyMOL 3.1.5.1 (Schrödinger, LLC, accessed March 2025).

## Supporting Information

Supporting information for this article is available on the WWW under https://doi.org/10.1002/cbdv.71532.

## Author Contributions

**Lorena C Albernaz**: conceptualization, methodology, formal analysis, investigation, writing – original draft. Alice M S Rodrigues and **Laila S Espindola**: methodology, formal analysis, resources and writing – review. **Lais S Morais, Rodrigo and Amanda M Alameida**: investigation. **Marion Girardot**: conceptualization, methodology, supervision and resources. **Christine Imbert**: methodology, validation, resources, writing – review & editing, supervision.

## Conflicts of Interest

The authors declare no conflicts of interest.

## Supporting information




**Supporting File 1**: cbdv71532‐sup‐0001‐SuppMat.docx

**Supporting File 2**: cbdv71532‐sup‐0002‐FigureSI1.docx

## Data Availability

The data that support the findings of this study are available from the corresponding author upon reasonable request.
