# Supplementary material for: Integrated Approach for the Discovery of Antifungal and Antibiofilm Agents From Cerrado Plants
Source: Chem Biodivers. 2026 Jul 26;23(7):e71532. doi: 10.1002/cbdv.71532 (PMC13401958; doi:10.1002/cbdv.71532)
Supplement: Supplementary file 1 — Supporting File 1: cbdv71532‐sup‐0001‐SuppMat.docx [file CBDV-23-e71532-s001.docx]

**Supplementary Material**

**Integrated Approach for the Discovery of Antifungal and Anti-biofilm Agents from Cerrado Plants**

Lorena C Albernaz^a*^, Amanda M Almeida ^a^, Laís S Morais ^a^, Alice M S Rodrigues^b^, Rodrigo S Ferreira ^a^**,** Laila S Espindola^a^, Marion Girardot^c^, Christine Imbert^c^

^a^Laboratório de Farmacognosia, Universidade de Brasília, Campus Universitário Darcy Ribeiro, Brasília 70910-900, Brazil

^b^Laboratoire de Biodiversité et Biotechnologies Microbiennes, UAR 3579, Université Sorbonne, Observatoire Oceanologique, Banyuls-sur-Mer, France

^c^Laboratoire Ecologie et Biologie des Interactions (EBI), UMR CNRS 7267, Université de Poitiers, Poitiers, France

Correspondence: Lorena C Albernaz (lalbernaz@unb.br)

| Table SM1. Inhibitory activity of extracts exhibiting less than 50% inhibition. | | | | |
| --- | --- | --- | --- | --- |
| **Sample** | **Plant Part** | **Solvent** | **% Inhibition ±SD** | |
|  |  |  | **Biofilm in formation** | **Mature Biofilm** |
| *Zeyheria montana* | root (bark) | ethyl_acetate | NA | NA |
|  | root (wood) | hexane | 45.88 ± 1.24 | 48.74 ± 0.33 |
|  | stem (bark+wood) | ethanol | NA | NA |
|  |  | ethyl_acetate | NA | 20.49 ± 4.16 |
|  |  | hexane | 26.08 ± 7.41 | NA |
|  | leaves + fruit | hexane | 23.08 ± 7.37 | 33.06 ± 2.89 |
| *Cheiloclinium cognatum* | stem (wood) | ethyl_acetate | 28.55 ± 10.35 | 27.49 ± 1.88 |
|  |  | ethanol | 32.54 ± 7.87 | 23.22 ± 9.54 |
|  |  | hexane | 29.58 ± 1.23 | 24.87 ± 1.62 |
|  | stem (bark) | hexane | NA | 21.81 ± 8.52 |
|  |  | water | 21.24 ± 1.05 | NA |
|  | leaves | hexane | NA | NA |
|  |  | ethyl_acetate | NA | NA |
| *Plenckia populnea* | stem (bark) | ethyl_acetate | 41.36 ± 5.62 | 40.53 ± 1.66 |
|  |  | ethanol | 44.49 ± 1.87 | NA |
|  | leaves | ethyl_acetate | 44.16 ± 2.18 | 24.47 ± 2.77 |
|  |  | ethanol | 28.38 ± 2.66 | NA |
|  | stem (wood) | hexane | NA | NA |
|  |  | ethyl_acetate | NA | 35.37 ± 1.86 |
| *Davilla elliptica* | leaves | ethyl_acetate | 25.54 ± 5.57 | NA |
|  |  | hexane | NA | NA |
|  |  | ethanol | 28.33 ± 7.49 | NA |
|  | stem (wood) | ethanol | 30.72 ± 2.44 | NA |
|  | stem (bark) | hexane | NA | NA |
| *Andira humilis* | root (bark) | ethanol | NA | NA |
|  | leaves | ethanol | 24.45 ± 5.48 | NA |
|  | root (wood) | hexane | 20.39 ± 3.89 | NA |
|  | stem (wood) | ethyl_acetate | NA | 39.58 ± 5.96 |
|  |  | ethanol | NA | 38.35 ± 4.66 |
|  | stem (bark) | ethanol | NA | 42.25 ± 4.02 |
|  | root (bark) | hexane | NA | 44.24 ± 2.54 |
| *Andira vermifuga* | leaves | hexane | NA | 29.40 ± 6.00 |
| *Chamaecrista desvauxii* | aerial parts | ethyl_acetate | 21.79 ± 16.39 | NA |
|  |  | ethanol | NA | NA |
|  |  | hexane | NA | NA |
| *Sclerolobium aureum* | stem (wood) | hexane | 20.08 ± 10.62 | 38.59 ± 7.46 |
|  | root (bark) | ethyl_acetate | 34.89 ± 6.46 | 41.13 ± 6.55 |
|  | leaves | ethyl_acetate | 34.72 ± 6.66 | 26.88 ± 6.37 |
| *Tachigali vulgaris* | leaves | ethyl_acetate | 37.27 ± 3.70 | 41.24 ± 2.24 |
|  |  | hexane | 33.78 ± 5.60 | 22.86 ± 5.87 |
|  | leaves | ethanol | 46.63 ± 1.37 | NA |
|  | stem (wood) | hexane | 39.60 ± 7.31 | 36.81 ± 2.20 |
|  | stem (bark) | hexane | 29.52 ± 8.32 | NA |
|  |  | ethyl_acetate | 36.56 ± 3.31 | 33.55 ± 5.33 |
| *Vatairea macrocarpa* | root (bark) | ethyl_acetate | 44.09±3.61 | NA |
|  | root (wood) | hexane | NA | NA |
|  | stem (bark) | ethyl_acetate | NA | NA |
|  | stem (wood) | ethanol | NA | NA |
|  |  | hexane | NA | NA |
| *Blepharocalyx salicifolius* | leaves | hexane | 21.32 ± 9.47 | NA |
|  |  | ethyl_acetate | 42.60 ± 5.81 | 40.66 ± 3.87 |
|  |  | water | NA | NA |
|  |  | ethanol | 41.39 ± 5.54 | 38.30 ± 7.40 |
|  | stem (bark) | hexane | 20.37 ± 9.31 | NA |
|  | root (bark) | water | 25.27 ± 1.46 | 24.60 ± 10.91 |
| *Eugenia dysenterica* | leaves | hydroalcoholic | 42.55 ± 4.61 | 40.99 ± 5.67 |
| *Neea theifera* | root (bark) | ethyl_acetate | 37.34 ± 5.93 | 29.36 ± 9.33 |
|  | root (wood) | ethyl_acetate | 41.26 ± 5.44 | 22.66 ± 9.05 |
|  |  | hexane | 34.95 ± 5.08 | NA |
|  |  | ethanol | NA | NA |
|  | stem (wood) | hexane | NA | NA |
|  |  | ethyl_acetate | 30.63 ± 8.55 | NA |
|  | leaves | ethyl_acetate | NA | NA |
| *Guapira noxia* | stem (wood) | ethyl_acetate | NA | NA |
|  | root (wood) | ethyl_acetate | NA | NA |
|  | root (bark) | ethyl_acetate | NA | NA |
| *Ouratea floribunda* | fruit | hexane | 30.06 ± 5.35 | 21.93 ± 4.22 |
|  | leaves | dichloromethane | NA | NA |
| *Agonandra brasiliensis* | leaves | hexane | NA | NA |
|  |  | ethanol | NA | 25.53 ± 5.07 |
|  |  | ethyl_acetate | NA | NA |
| *Chrysophyllum soboliferum* | leaves | ethanol | NA | NA |
|  |  | hexane | NA | NA |
| *Symplocos rhamnifolia* | stem (bark) | ethyl_acetate | 38.56 ± 6.76 | 25.57 ± 8.35 |
|  | stem (wood) | ethyl_acetate | NA | NA |
|  | root (bark) | ethyl_acetate | NA | NA |
|  | root (wood) | ethyl_acetate | NA | NA |
| *Lippia rotundifolia* | leaves | ethanol | NA | 27.62 ± 7.71 |
|  |  | ethyl_acetate | 30.52 ± 6.05 | 43.83 ± 2.60 |
|  |  | hexane | 29.32 ± 11.14 | 39.12 ± 3.59 |
|  | flower | hexane | 42.04 ± 5.38 | 28.69 ± 5.77 |
|  |  | ethyl_acetate | 45.51 ± 1.71 | 43.82 ± 1.51 |
|  | stem (wood) | hexane | 21.71 ± 7.59 | 25.13 ± 8.73 |
|  |  | ethyl_acetate | 28.36 ± 6.45 | 38.65 ± 6.94 |
|  | stem (bark) | ethyl_acetate | 35.64 ± 4.01 | 41.32 ± 2.37 |
|  | root (wood + bark) | hexane | 39.24 ± 7.54 | NA |
| *Salvertia convallariodora* | stem (wood) | ethanol | 32.04 ± 10.32 | 42.41 ± 5.56 |
|  |  | ethyl_acetate | 40.56 ± 7.45 | 43.60 ± 4.45 |
|  | leaves | ethyl_acetate | 21.93 ± 3.94 | NA |
|  |  | ethanol | NA | NA |
| NA: inhibition ≤ 20% | | | | |

Table SI2A: Evaluation of anti-development activity against *C. albicans* clinical strains (S1 and S3), and bioactive score.

| **Samples** | | **% Inhibition±DP (IC 95%)** | | **Bioactive Score** | |
| --- | --- | --- | --- | --- | --- |
|  |  | **S1** | **S3** | **S1** | **S3** |
| *Andira humilis* | RB EA | 52.74±16.72 (43.28-62.20) | 36.02±5.36 (32.99-39.05) | 48 (L) | 38 (L) |
| *Blepharocalyx salicifolius* | SW EA | 42.48±14.80 (34.11-50.85) | 86.61±8.38 (81.87-91.35) | 37 (L) | 97 (H) |
|  | SB EA | 62.05±8.46 (57.26-66.84) | 75.50±16.51 (66.16-84.84) | 72 (H) | 73 (H) |
| *Cheiloclinium cognatum* | L E | 82.17±7.79 (77.76-86.58) | 87.62±6.98 (83.67-91.57) | 100 (H) | 100 (H) |
| *Hymenaea stigonocarpa* | SW H | 46.57±9.25 (41.34-51.08) | 37.32±6.27 (37.77-40.87) | 49 (L) | 39 (L) |
|  | L H | 56.65±5.71 (53.42-59.88) | 50.94±7.66 (46.61-55.27) | 68 (H) | 54 (L) |
|  | L EA | 58.53±15.40 (49.82-67.24) | 43.14±5.39 (40.09-46.19) | 58 (L) | 47 (L) |
| *Myrcia linearifolia* | R EA | 48.11±16.02 (39.05-57.17) | 62.06±10.45 (56.15-67.97) | 43 (L) | 64 (H) |
|  | AP EA | 61.03±13.93 (53.15-68.91) | 68.03±8.40 (63.28-72.78) | 63 (H) | 74 (H) |
| *Sclerolobium aureum* | RW E | 49.27±10.26 (43.46-55.08) | 39.01±9.35 (33.72-44.30) | 52 (L) | 37 (L) |
|  | SW E | 55.10±12.50 (48.03-62.17) | 42.60±12.64 (35.45-49.75) | 57 (L) | 37 (L) |
|  | SW EA | 72.84±15.42 (64.12-81.56) | 57.42±8.60 (52.55-62.29) | 77 (H) | 61 (H) |
|  | SB EA | 37.26±6.86 (33.38-41.14) | 30.41±4.41 (27.91-32.91) | 41 (L) | 32 (L) |
|  | RW H | 15.60±2.38 (14.25-16.95) | 13.22±1.91 (12.14-14.30) | 18 (L) | 14 (L) |
|  | RW EA | 74.74±15.55 (65.94-83.54) | 59.20±9.71 (53.71-64.69) | 80 (H) | 61 (H) |
| *Tachigali vulgaris* | SW EA | 72.04±9.21 (66.83-77.25) | 62.83±8.53 (58-67.66) | 84 (H) | 67 (H) |
| *Vatairea macrocarpa* | RW EA | 19.25±3.43 (17.31-21.19) | 15.81±1.72 (14.84-16.78) | 21 (L) | 17 (L) |
|  | L EA | 0 | 0 | 0 | 0 |
| R: root; RB: root bark; RW: root wood; S: stem; SB: stem bark; SW: stem wood; L: leaves; AP: aerial parts; H: Hexane; EA: ethyl acetate; E: ethanol; L: Low; H: High | | | | | |

Table SI2B: Evaluation of Anti-biofilm activity against *C. albicans* clinical strains (S1 and S3), and bioactive score.

| **Samples** | | **% Inhibition±DP (IC 95%)** | | **Bioactive Score** |
| --- | --- | --- | --- | --- |
|  |  | **S1** | **S3** | **S1** |
| *Andira humilis* | RB EA | 24.99±6.12 (21.53-28.45) | 27.24±3.11 (25.48-29.0) | 27 (L) |
| *Blepharocalyx salicifolius* | SW EA | 58.49±14.34 (50.38-66.6) | 24.80±2.29 (23.5-26.1) | 64 (H) |
|  | SB EA | 73.41±12.05 (66.59-80.23) | 18.43±1.11 (17.8-19.06) | 89 (H) |
| *Cheiloclinium cognatum* | L E | 77.23±15.47 (68.48-85.98) | 40.62±6.26 (37.08-44.16) | 90 (H) |
| *Hymenaea stigonocarpa* | SW H | 18.28±2.60 (16.81-19.75) | 21.62±3.69 (19.53-23.71) | 23 (L) |
|  | L EA | 74.36±5.59 (71.2-77.52) | 32.54±2.76 (30.98-34.1) | 100 (H) |
|  | L H | 62.88±16.69 (53.44-72.32) | 26.88±3.46 (24.92-28.84) | 67 (H) |
| *Myrcia linearifolia* | R EA | 63.06±6.21 (59.55-66.57) | 21.11±1.00 (20.54-21.68) | 82 (H) |
|  | AP EA | 64.90±13.86 (57.06-72.74) | 27.45±1.60 (26.54-28.36) | 74 (H) |
| *Sclerolobium aureum* | RW H | 17.68±0.88 (17.18-18.18) | 19.01±1.91 (17.93-20.09) | 24 (L) |
|  | RW E | 31.86±7.36 (27.7-36.02) | 15.42±2.58 (13.96-16.88) | 36 (L) |
|  | RW EA | 33.46±6.66 (29.69-37.23) | 14.35±3.4 (12.43-16.27) | 39 (L) |
|  | SW E | 29.92±5.73 (26.68-33.16) | 3.68±1.01 (3.11-4.25) | 35 (L) |
|  | SW EA | 27.56±2.83 (25.96-29.16) | 9.15±1.73 (8.17-10.13) | 36 (L) |
|  | SB EA | 27.20±3.39 (25.28-29.12) | 17.99±3.11 (16.23-19.75) | 35 (L) |
| *Tachigali vulgaris* | SW EA | 61.95±11.24 (55.59-68.31) | 27.67±4.31 (25.23-30.11) | 74 (H) |
| *Vatairea macrocarpa* | RW EA | 22.47±3.43 (20.53-24.41) | 30.18±4.72 (27.51-32.85) | 28 (L) |
|  | L EA | 18.29±1.16 (17.63-18.95) | 29.55±7.68 (25.2-33.9) | 25 (L) |
| R: root; RB: root bark; RW: root wood; S: stem; SB: stem bark; SW: stem wood; L: leaves; AP: aerial parts; H: Hexane; EA: ethyl acetate; E: ethanol; L: Low; H: High | | | | |

Table SI3: Concordance analysis of Bioactive Score (BS) thresholds (≥50, ≥60, ≥70).

Percent agreement and Cohen’s κ values between cutoffs across datasets.

| BS | Comparison | N | Percent_Agreement | Kappa |
| --- | --- | --- | --- | --- |
| BS_S1 | 60 vs 50 | 18 | 0.8333333333333334 | 0.674698795180723 |
| BS_S1 | 60 vs 70 | 18 | 0.8888888888888888 | 0.7534246575342465 |
| BS_S3 | 60 vs 50 | 18 | 0.9444444444444444 | 0.8888888888888888 |
| BS_S3 | 60 vs 70 | 18 | 0.7777777777777778 | 0.5263157894736842 |
| BS_S1_Mature | 60 vs 50 | 18 | 1.0 | 1.0 |
| BS_S1_Mature | 60 vs 70 | 18 | 0.8888888888888888 | 0.769230769230769 |
| BS_agg | 60 vs 50 | 18 | 0.8333333333333334 | 0.674698795180723 |
| BS_agg | 60 vs 70 | 18 | 0.8888888888888888 | 0.7534246575342465 |

Table SI4: Correlation of Bioactive Score (BS) with antifungal endpoints.

Spearman’s ρ and p-values for correlations between BS values and MIC or hyphal inhibition assays.

| BS | Endpoint | n | Spearman_rho | p_value | Expectation |
| --- | --- | --- | --- | --- | --- |
| BS_S1 | MIC_ATCC28367 | 18 | -0.3210162560387533 | 0.1939841520309601 | negative (higher BS, lower MIC) |
| BS_S1 | MIC_ATCC10231 | 18 | -0.1049096771137555 | 0.6786641366022753 | negative (higher BS, lower MIC) |
| BS_S1 | MIC_S1 | 13 | -0.0196678879867742 | 0.949150662405322 | negative (higher BS, lower MIC) |
| BS_S1 | MIC_S3 | 13 | -0.043411385355121 | 0.8880159408108984 | negative (higher BS, lower MIC) |
| BS_S1 | MIC_Tropicalis | 13 | 0.1148680220095078 | 0.7086483710794858 | negative (higher BS, lower MIC) |
| BS_S1 | MIC_Krusei | 13 | 0.0028056700404862 | 0.9927420995760172 | negative (higher BS, lower MIC) |
| BS_S1 | Hyphal_inhibition | 18 | 0.1064268627403085 | 0.6742573582195649 | positive (higher BS, higher inhibition) |
| BS_S3 | MIC_ATCC28367 | 18 | -0.0928570680937984 | 0.7140146027631683 | negative (higher BS, lower MIC) |
| BS_S3 | MIC_ATCC10231 | 18 | 0.0880454869308294 | 0.7282879793264865 | negative (higher BS, lower MIC) |
| BS_S3 | MIC_S1 | 13 | 0.1732731252076406 | 0.5713214096469574 | negative (higher BS, lower MIC) |
| BS_S3 | MIC_S3 | 13 | 0.2162046676046344 | 0.4780445498291472 | negative (higher BS, lower MIC) |
| BS_S3 | MIC_Tropicalis | 13 | 0.455120824048989 | 0.1181213737948758 | negative (higher BS, lower MIC) |
| BS_S3 | MIC_Krusei | 13 | 0.4585858030419202 | 0.1149954343664649 | negative (higher BS, lower MIC) |
| BS_S3 | Hyphal_inhibition | 18 | -0.0397525614533527 | 0.8755531375831167 | positive (higher BS, higher inhibition) |
| BS_S1_Mature | MIC_ATCC28367 | 18 | 0.0678520527102187 | 0.7890741362616749 | negative (higher BS, lower MIC) |
| BS_S1_Mature | MIC_ATCC10231 | 18 | 0.2117369149945412 | 0.3989729479353034 | negative (higher BS, lower MIC) |
| BS_S1_Mature | MIC_S1 | 13 | 0.2335259555862925 | 0.4425695640533609 | negative (higher BS, lower MIC) |
| BS_S1_Mature | MIC_S3 | 13 | 0.4274662053492836 | 0.145121573349245 | negative (higher BS, lower MIC) |
| BS_S1_Mature | MIC_Tropicalis | 13 | 0.5274369053249687 | 0.0639761411752234 | negative (higher BS, lower MIC) |
| BS_S1_Mature | MIC_Krusei | 13 | 0.4593584724987274 | 0.1143060312798582 | negative (higher BS, lower MIC) |
| BS_S1_Mature | Hyphal_inhibition | 18 | 0.0906827409396103 | 0.7204537959430447 | positive (higher BS, higher inhibition) |
| BS_agg | MIC_ATCC28367 | 18 | -0.1506844252013462 | 0.5506222363977868 | negative (higher BS, lower MIC) |
| BS_agg | MIC_ATCC10231 | 18 | 0.0758072264507448 | 0.7649680134939811 | negative (higher BS, lower MIC) |
| BS_agg | MIC_S1 | 13 | 0.1097290634682579 | 0.7212042766596068 | negative (higher BS, lower MIC) |
| BS_agg | MIC_S3 | 13 | 0.2072124317455849 | 0.4969688757286477 | negative (higher BS, lower MIC) |
| BS_agg | MIC_Tropicalis | 13 | 0.4488824726169947 | 0.1238918858616318 | negative (higher BS, lower MIC) |
| BS_agg | MIC_Krusei | 13 | 0.4396917489055096 | 0.1327310182947517 | negative (higher BS, lower MIC) |
| BS_agg | Hyphal_inhibition | 18 | 0.0333748991517645 | 0.8954050782923149 | positive (higher BS, higher inhibition) |

Table SI5: Antiplanktonic activity against *Candida* sp.

| **Plant** | **Parts of plant (solvents)** | **MIC (µg/mL)** | | | | | |
| --- | --- | --- | --- | --- | --- | --- | --- |
|  |  | ***C. albicans* (ATCC** 28367**)** | ***C. albicans* (ATCC10231)** | ***C. albicans* (S1)** | ***C. albicans* (S3)** | ***C. tropicalis* (LMGO 49)** | ***C. krusei* (LMGO 174)** |
| *Andira humilis* | RB EA | 15.625 | 3.9 | 15.625 | 3.9 | 3.9 | 3.9 |
| *Blepharocalyx salicifolius* | SW EA | 15.625 | 15.625 | 15.625 | 7.81 | 7.81 | 15.625 |
|  | SB EA | 7.81 | 3.9 | 7.81 | 7.81 | 7.81 | 7.81 |
| *Cheiloclinium cognatum* | L E | 62.5 | 62.5 | 31.25 | 62.5 | 15.625 | 15.625 |
| *Hymenaea stigonocarpa* | SW H | > 1000 | > 1000 | ND | ND | ND | ND |
|  | L H | 500 | 1000 | ND | ND | ND | ND |
|  | L EA | 31.25 | 3.9 | 31.25 | 15.625 | 7.81 | 7.81 |
| *Myrcia linearifolia* | R EA | 7.81 | 3.9 | 7.81 | 7.81 | 7.81 | 15.625 |
|  | AP EA | >1000 | > 1000 | ND | ND | ND | ND |
| *Sclerolobium aureum* | RW E | 0.97 | 0.49 | 0.97 | 0.97 | 0.49 | 1.95 |
|  | SW E | 3.9 | 0.97 | 0.97 | 0.97 | 0.97 | 1.95 |
|  | SW EA | 3.9 | 0.97 | 15.625 | 7.81 | 0.97 | 1.95 |
|  | SB EA | 7.81 | 0.97 | 7.81 | 7.81 | 0.97 | 0.97 |
|  | RW H | > 1000 | > 1000 | ND | ND | ND | ND |
|  | RW EA | 3.9 | 1.95 | 1.95 | 1.95 | 1.95 | 1.95 |
| *Tachigali vulgaris* | SW EA | 7.81 | > 1000 | 15.625 | 7.81 | >1000 | >1000 |
| *Vatairea macrocarpa* | RW EA | 250 | > 1000 | ND | ND | ND | ND |
|  | L EA | 125 | 62.5 | 125 | 125 | 31.25 | 31.25 |
| R: root; RB: root bark; RW: root wood; SB: stem bark; SW: stem wood; L: leaves; AP: aerial parts; H: Hexane; EA: ethyl acetate; E: ethanol; ND: Not Determinate | | | | | | | |

Table SI6: Molecular networking and *in silico*annotation of compounds in bioactive Cerrado extracts.

| **Compound Name** | **Adduct** | **Molecular Formula** | **Fragmentation (%)** | **Experimental *m/z*** | **Rt (min)** | **Error (ppm)** | **Similarity MS/MS (Library GNPS)** | **CSI:FingerID Score** | **Extract** |
| --- | --- | --- | --- | --- | --- | --- | --- | --- | --- |
| **Flavonoids** | | | | | | | | | |
| Epicatechin | [M^+^H]^+^ | C_15_H_14_O_6_ | 139.0394 (100), 123.0442 (75), 165.0546 (12.5) | 291.0872 | 08.63 | 1.37 | GNPS-LIBRARY | -8,061 | ML REA |
| Catechin | [M^+^H]^+^ | C_15_H_14_O_6_ | 139.0393 (100); 123.0442 (81.5); 147.0439 (13.7) | 291.0876 | 10.69 | 2.40 | Private-LIB | -8.832 | SA RWE |
| Epicatechin-3-gallate | [M^+^H]^+^ | C_22_H_18_O_10_ | 123.0443 (100); 139.0391 (60); 153.0188 (13.5) | 443.0989 | 11.97 | 2.48 | GNPS-NIST14 | -37.369 | SA SWEA |
| Calycosin | [M^+^H]^+^ | C_16_H_12_O_5_ | 285.0760 (100); 213.0545 (20.7); 137.0229 (14) | 285.0759 | 14.21 | 1.40 | BMDMS-NP | -43.422 | VM LEA  AH RBEA |
| Formononetin | [M^+^H]^+^ | C_16_H_12_O_4_ | 269.0804 (100); 197.0589 (17.4); 225.0538 (6.7) | 269.0811 | 16.19 | 1.11 | MONA | -36.549 | VM LEA  VM RWEA |
| 7,4-Methoxy-3-hydroxyflavone | [M^+^H]^+^ | C_17_H_14_O_5_ | 299.0915 (100); 227.0703 (19.65); 151.0386 (12.47) | 299.0915 | 16.34 | 1.34 | - | -72.014 | AH RBEA |
| 5,3'-Dihydroxy-3,8,4',5'-tetramethoxy-6,7-methylenedioxyflavone | [M^+^H]^+^ | C_20_H_18_O_10_ | 419.0983 (100); 404.0747 (10.98); 389.0516 (9.07) | 419.0982 | 16.58 | 1.20 | - | -66.315 | ML REA  ML APEA |
| 3,5-Dihydroxy-6,7,8-trimethoxy-3',4'-methylenedioxyflavone | [M^+^H]^+^ | C_19_H_16_O_9_ | 389.0881 (100); 331.0457 (28.63); 374.0644 (17.90) | 389.0879 | 16.73 | 2.05 | - | -65.703 | ML REA  ML APEA |
| Prunetin | [M^+^H]^+^ | C_16_H_12_O_5_ | 285.0756 (100); 242.0564 (9); 167.0333 (5) | 285.0771 | 17.69 | 2.80 | BMDMS-NP | -22.269 | VM LEA |
| Biochanin A | [M^+^H]^+^ | C_16_H_12_O_5_ | 285.0765 (100); 213.0544 (12); 124.0145 (5) | 285.0764 | 17.72 | 0.35 | MONA | -38.259 | AH RBEA |
| Tricin | [M^+^H]^+^ | C_17_H_14_O_7_ | 331.0817 (100); 315.0503 (28.6); 258.0522 (10.9) | 331.0817 | 15.33 | 0.30 | Massbank | -31.350 | HS LEA |
| Chryseriol | [M^+^H]^+^ | C_16_H_12_O_6_ | 301.0697 (100); 229.0490 (20.75); 153.0170 (9.63) | 301.0702 | 15.48 | 3.32 | MONA | -43.361 | VM LEA |
| Isokaempferide | [M^+^H]^+^ | C_16_H_12_O_6_ | 301.0720 (100); 229.0491 (22.83); 153.0175 (11.1) | 301.0721 | 15.50 | 2.99 | MONA | -43.121 | AH RBEA |
| Erythrinin C | [M^+^H]^+^ | C_20_H_18_O_6_ | 283.0600 (100); 355.1180 (39.4); 165.0178 (12.08) | 355.1179 | 13.26 | 0.56 | - | -75.886 | VM LEA |
| Pseudobaptigenin | [M^+^H]^+^ | C_16_H_10_O_5_ | 283.0605 (100); 225.0541 (18.7); 197.0595 (13.5) | 283.0606 | 16.04 | 0 | GNPS –NIH | - | AH RBEA |
| Medicarpin | [M^+^H]^+^ | C_16_H_14_O_4_ | 137.0597 (100); 109.0646 (13); 123.0438 (8) | 271.0960 | 17.11 | 3.69 | GNPS-NIST14 | -25.209 | AH RBEA |
| Pseudobaptigenin Methyl Ether | [M^+^H]^+^ | _C17_H_12_O_5_ | 297.0765 (100); 196.0511 (22.55); 224.0464 (16.03) | 297.0763 | 18.33 | 0.68 | - | -80.238 | AH RBEA |
| Lupiwighteone | [M^+^H]^+^ | C_20_H_18_O_5_ | 283.0598 100); 79.0163 (30.53); 241.0494 (17.61) | 339.1218 | 18.61 | 4.13 | - | -32.556 | VM LEA |
| Procyanidin B2 | [M^+^H]^+^ | C_30_H_26_O_12_ | 127.0393 (100); 289.0711 (64.4); 409.0919 (28.9); 163.0390 (35.6) | 579.1520 | 09.85 | 2.93 | GNPS-NIST14 | -9.915 | SA SBEA |
| Luteolin | [M^+^H]^+^ | C_15_H_10_O_6_ | 287.0552 (100); 153.0180 (14.4); 135.0439 (5.9) | 287.0551 | 14.18 | 1.74 | BMDMS-NP | -6.530 | HS LEA |
| **Glycosylated flavonoids** | | | | | | | | | |
| Myricetin 3-galactoside | [M^+^H]^+^ | C_21_H_20_O_13_ | 319.0450 (100); 153.0177 (5.1); 85.0277 (3.1) | 481.0991 | 9.55 | 1.87 | Respect | -19.396 | ML APEA |
| Myricetin 3-xyloside | [M^+^H]^+^ | C_20_H_18_O_12_ | 319.0451 (100); 153.0181 (5.4); 73.0279 (0.8) | 451.0874 | 09.93 | 0.66 | Respect | -16.640 | ML APEA |
| Myricetin 3-rhamnoside | [M^+^H]^+^ | C_21_H_20_O_12_ | 319.0454 (100); 85.0281 (12); 153.0183 (4.1) | 465.1037 | 10.01 | 0.86 | Massbank | -15.815 | ML APEA |
| Lepidoside | [M^+^H]^+^ | C_26_H_28_O_14_ | 287.0555 (100); 288.0587 (15.3); 419.0974 (9.4) | 565.1557 | 10.62 | 0 | GNPS –NIH | -27.477 | ML APEA |
| Quercetin 3-rhamnoside | [M^+^H]^+^ | C_21_H_20_O_11_ | 303.0505 (100); 85.0280 (11.9); 129.0546 (10.5) | 449.1088 | 10.77 | 0.44 | Massbank | -21.599 | ML APEA |
| Myricetin 3-(2”-acetylrhamnoside) | [M^+^H]^+^ | C_23_H_22_O_13_ | 303.0491 (100); 187.0602 (9.3); 127.0383 (5.9) | 507.1150 | 11.07 | 0.197 | GNPS –NIH | -51.595 | ML APEA |
| Rutin | [M^+^H]^+^ | C_27_H_30_O_16_ | 303.0497 (100); 465.1020 (13.2); 129.0538 (3) | 611.1625 | 11.5 | 2.12 | GNPS-NIST14 | -18.719 | VM LEA |
| Quercetin 3-glucoside | [M^+^H]^+^ | C_21_H_20_O_12_ | 303.0495 (100); 304.0526 (14.8); 85.0268 (3.5) | 465.1024 | 11.81 | 1.94 | Massbank | -19.381 | VM LEA |
| Narcissin | [M^+^H]^+^ | C_28_H_32_O_16_ | 317.0656 (100); 318.0689 (15.9); 479.1179 (10) | 625.1775 | 11.89 | 0.96 | GNPS-NIST14 | -21.037 | HS LEA |
| Nicotiflorine | [M^+^H]^+^ | C_27_H_30_O_15_ | 287.0544 (100); 301.0700 (71.4); 463.1230 (28.2) | 595.1660 | 11.96 | 0.50 | Massbank | -85.551 | VM LEA |
| Quercetin 3-O-(6''-acetyl-glucoside) | [M^+^H]^+^ | C_23_H_22_O_13_ | 303.0491 (100); 304.0530 (14.4); 187.0602 (9.3) | 507.1133 | 12.50 | 1.18 | GNPS –NIH- | -7.020 | VM LEA |
| Engeletin | [M^+^H]^+^ | C_21_H_22_O_10_ | 85.0278 (100); 129.0544 (92.72); 289.0707 (79.91) | 435.1299 | 12.52 | 1.84 | - | -58.586 | AH RBEA |
| Afzelin | [M^+^H]^+^ | C_21_H_20_O_10_ | 287.0551 (100); 288.0588 (12.6); 85.0278 (11.5) | 433.1144 | 12.88 | 2.08 | GNPS –NIH | -29.721 | HS LEA |
| Vitexin | [M^+^H]^+^ | C_21_H_20_O_10_ | 287.0551 (100); 85.0278 (11.5); 129.0543 (9.1) | 433.1134 | 10.01 | 0.23 | GNPS –NIH | -25.392 | CC LEA |
| **Terpenes** | | | | | | | | | |
| Loliolide | [M^+^H]^+^ | C_11_H_16_O_3_ | 179.1070 (100); 133.1012 (66); 107.0850 (46.7) | 197.1176 | 11.23 | 1.01 | GNPS | >100 | ML APEA  CC LE |
| 3-hydroxyadamantane-1-carboxylic Acid | [M^+^H]^+^ | C_11_H_16_O_3_ | 179.1066 (100); 133.1004 (60.32); 107.0845 (46.15) | 197.1175 | 12.88 | 1.5 | - | -84.648 | VM LEA |
| Castanogenin | [M^+^H]^+^ | C_30_H_46_O_6_ | 467.3158 (100); 449.3065 (9.26); 437.3051 (7.12) | 503.3361 | 15.28 | 2.18 | - | -51.969 | ML APEA |
| Diospyric Acid B | [M^+^H]^+^ | C_30_H4_6_O_6_ | 467.3166 (100); 449.3102 (7.88); 437.3035 (6.64) | 503.3387 | 15.35 | 2.78 | - | -47.553 | ML REA |
| Madecassic Acid | [M-H4O2+H]+ | C_30_H_48_O_6_ | 451.3198 (100); 119.0860 (85.95); 133.1010 (81.88) | 469.3306 | 15.58 | 3.69 | BERKELEY-LAB | -18.333 | BS SBEA |
| Arjugenin | [M-H2O+H]+ | C_30_H_48_O_6_ | 451.3220 (100); 133.1013 (99); 119.0860 (90.81) | 487.3425 | 15.66 | 0.41 | - | -20.536 | BS SWEA  ML REA |
| Arjunic Acid | [M^+^H]^+^ | C_30_H_48_O_5_ | 453.3356 (100); 201.1626 (31); 425.3398 (91) | 489.3567 | 17.57 | 2.66 | GNPS –NIH | -36.281 | BS SBEA |
| Nivenolide | [M^+^H]^+^ | C_20_H_28_O_4_ | 287.1990 (100); 269.1873 (21.9); 121.0999 (15.7) | 333.2036 | 17.90 | 9 | GNPS –NIH | -38.725 | HS LH |
| Kauradienoic Acid | [M- H_2_O +H]^+^ | C_18_H_30_O_2_ | 301.2154 (100); 119.0850 (76); 255.2095 (55) | 301.2151 | 18.66 | 4.64 | GNPS | -40.640 | HS LH |
| 15-hydroxy-kauradienoic Acid | [M^+^H]^+^ | C_20_H_28_O_3_ | 207.1379 (100); 299.1983 (31); 253.1934 (25) | 317.2105 | 19.58 | 4.7 | GNPS –NIH | -68.694 | HS LH |
| Sumaresinolic Acid | [M+H-H_2_O]^+^ | C_30_H_48_O_4_ | 437.3421 (100); 111.0807 (82); 189.1643 (65) | 455.3529 | 20.09 | 0.88 | BERKELEY-LAB | >100 | BS SWEA  ML REA |
| Ent-15-Oxo-16-kauren-19-oic Acid | [M^+^H]^+^ | C_20_H_28_O_3_ | 271.2038 (100); 299.1983 (31); 253.1934 (25) | 317.2102 | 20.11 | 3.78 | GNPS –NIH | >100 | HS LH |
| **Stilbenes** | | | | | | | | | |
| Resveratrol | [M^+^H]^+^ | C_14_H_12_O_3_ | 135.0443 (100); 107.0491 (94.4); 229.0865 (94.4); 119.0494 (45.6) | 229.0865 | 10.16 | 0 | Massbank | -8.631 | ML REA |
| 5,4'-Dihydroxy-3,4,3'-trimethoxybibenzyl | [M^+^H]^+^ | C_17_H_20_O_5_ | 137.0595 (100); 123.0438 (88.45); 241.0858 (57.44) | 305.1381 | 13.29 | 3.28 | - | -82.888 | TV SWEA |
| **Xanthones** | | | | | | | | | |
| Mangiferin | [M^+^H]^+^ | C_19_H_18_O_11_ | 273.0395 (100), 303.0507 (73.5), 327.0510 (38.8), 387.0714 (36.7) | 423.0928 | 08.78 | 0.2 | MSNLIB-POSITIVE | -11.256 | CC LEA  BS SBEA |
| 3-O-Methylmangiferin | [M^+^H]^+^ | C_20_H_20_O_11_ | 287.0558 (100); 317.0665 (52.8); 401.0879 (44.4) | 437.1091 | 09.17 | 1.6 | MSNLIB-POSITIVE | -26.164 | CC LEA |
| Isomangiferin | [M^+^H]^+^ | C_19_H_18_O_11_ | 273.0403 (100); 303.0506 (79.2); 327.0517 (39.6) | 423.0927 | 10.08 | 0 | GNPS –NIH | -45.947 | CC LEA |
| **Naphthoquinone** | | | | | | | | | |
| Dianellidin | [M^+^H]^+^ | C_13_H_12_O_3_ | 199.0751 (100); 128.0614 (62.74); 171.0803 (29.53) | 217.0857 | 12.50 |  | - | > 100 | VM RWEA |
| **Lipids** | | | | | | | | | |
| Glyceryl Palmitate | [M^+^H]^+^ | C_19_H_38_O_4_ | 313.2727 (100); 239.2363 (77); 95.0845 (52) | 331.2835 | 19.83 | 3.92 | GNPS –NIH | >100 | SA RWEA |
| Outros fenois | | | | | | | | | |
| Homovanillyl Alcohol 4-O-glucoside | [M+NH4]+ | C_15_H_22_O_8_ | 151.0759 (100); 119.0495 (18.3); 91.0540 (15.9) | 348.1657 | 08.33 | 0.86 | GNPS –NIH | -43.496 | ML REA |
| 3,4,5-trihydroxybenzoic acid | [M^+^H]^+^ | C_7_H_6_O_5_ | 109.0281 (100); 153.0176 (84.2); 125.0227 (68.4); 81.0332 (48.4) | 171.0298 | 06.95 | 4.09 | MONA | -13.527 | ML REA |
| Ethyl 3,4,5-trihydroxybenzoate | M^+^H | C9H10O5 | 127.0397 (100); 153.0190 (84.6); 109.0292 (61.5) | 199.0609 | 11.84 | 1.50 | Berkeley-LAB | -13.215 | SA RWEA |
| 3,3’,4- tri-O-methylflavellagic Acid | [M^+^H]^+^ | C_17_H_12_O_9_ | 361.0555 (100); 346.0327 (10); 303.0144 (9.8) | 361.0555 | 11.92 | 1.38 | - | -65.516 | ML REA |
| Ellagic Acid | [M^+^H]^+^ | C_14_H_6_O_8_ | 303.0137 (100); 201.0176 (18.9); 173.0231 (10.7) | 303.0137 | 10.31 | 1.32 | BMDMS-NP | -8.029 | BS SWEA |
| Chlorogenic Acid | [M^+^H]^+^ | C_16_H_18_O_9_ | 163.0390 (100), 135.0439 (26); 117.0334 (14) | 355.1035 | 08.55 | 1.69 | GNPS-NIST14 | - | ML APEA |
