# Supplementary figures and images for: Integrated Approach for the Discovery of Antifungal and Antibiofilm Agents From Cerrado Plants
Source: Chem Biodivers. 2026 Jul 26;23(7):e71532. doi: 10.1002/cbdv.71532 (PMC13401958; doi:10.1002/cbdv.71532)

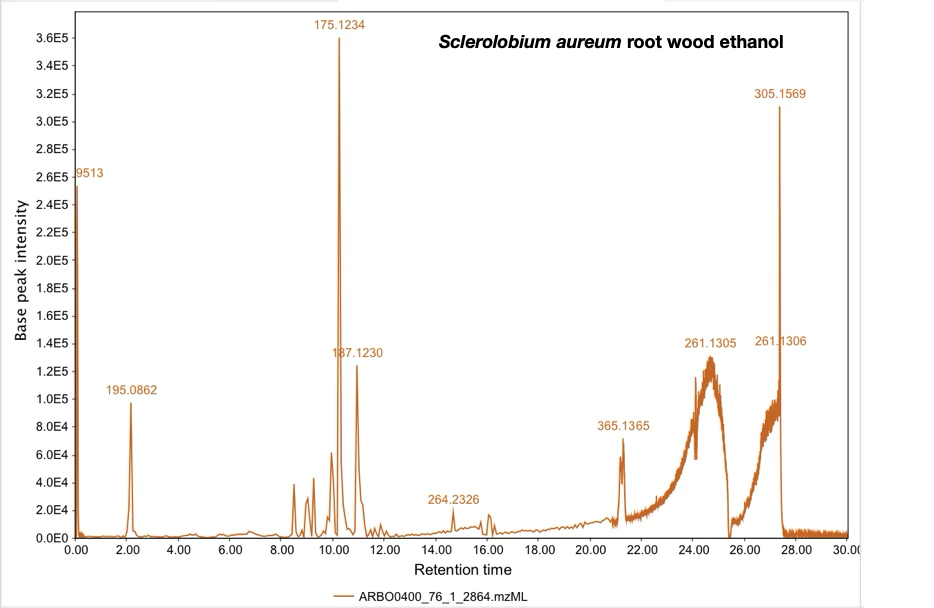


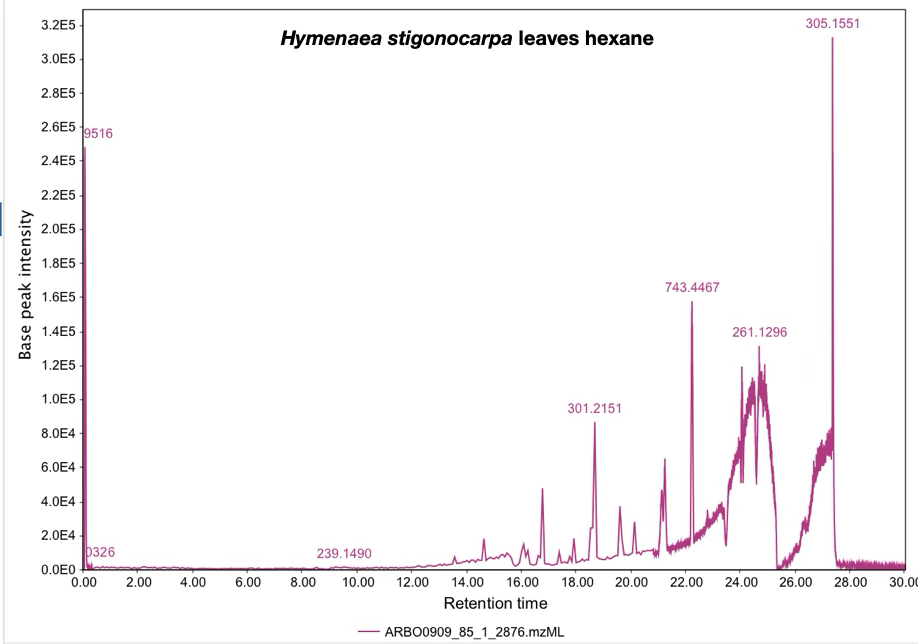


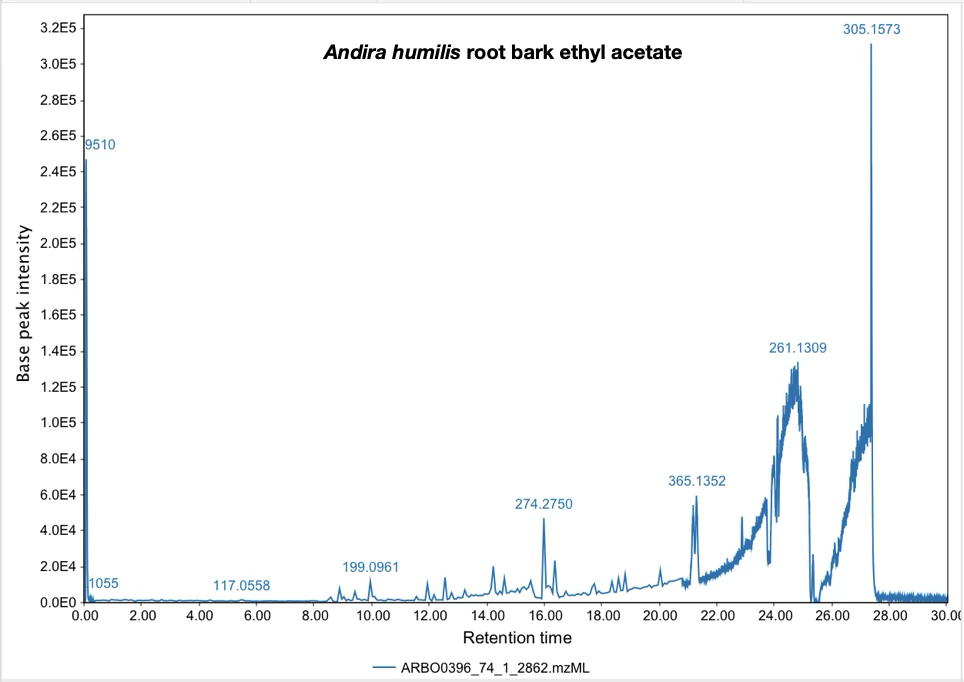


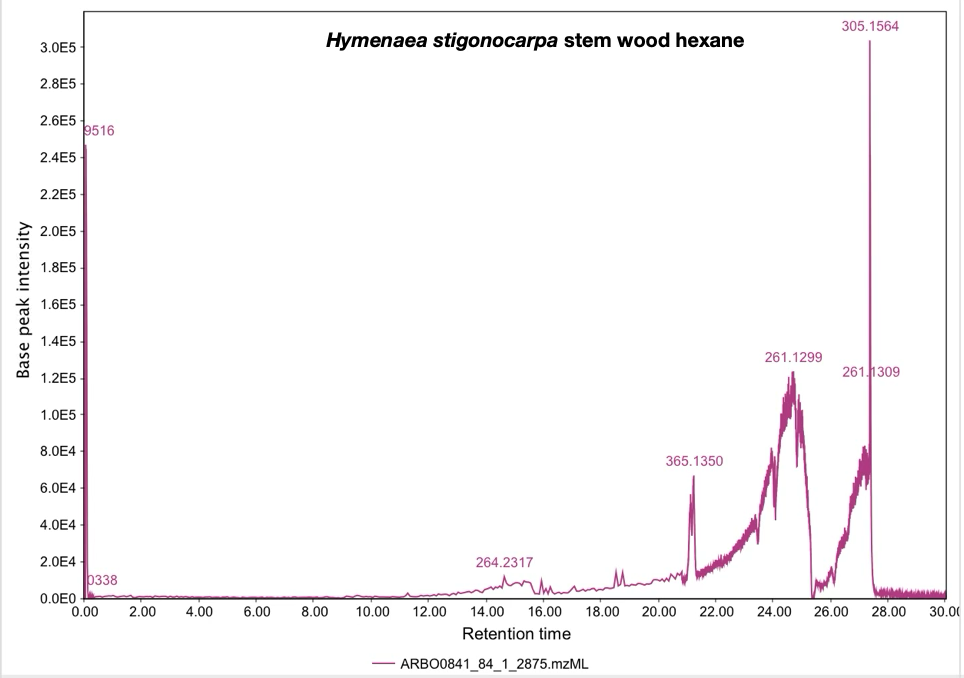


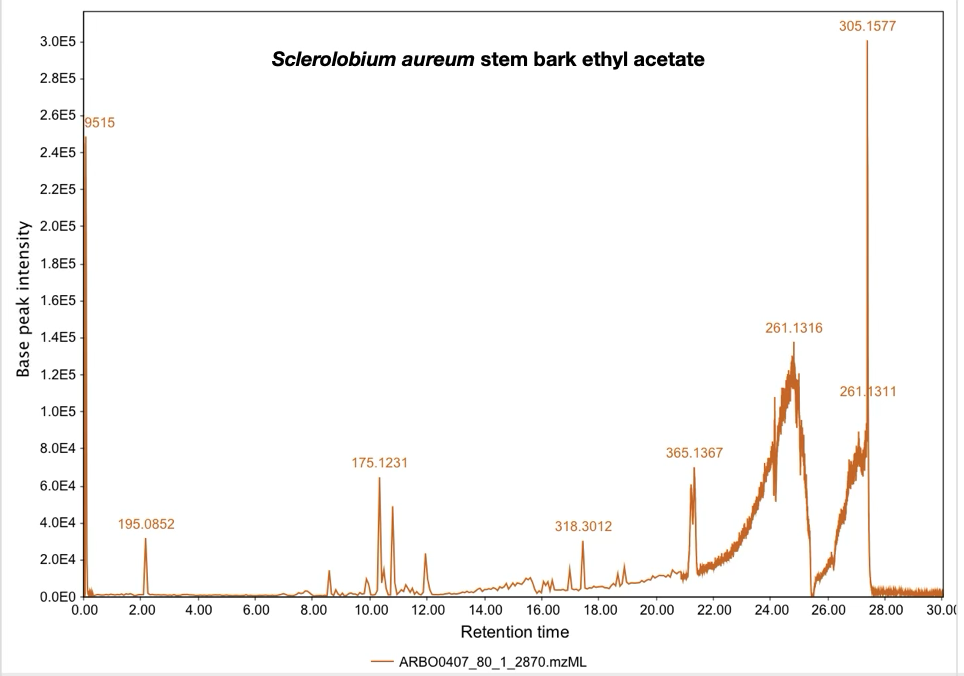


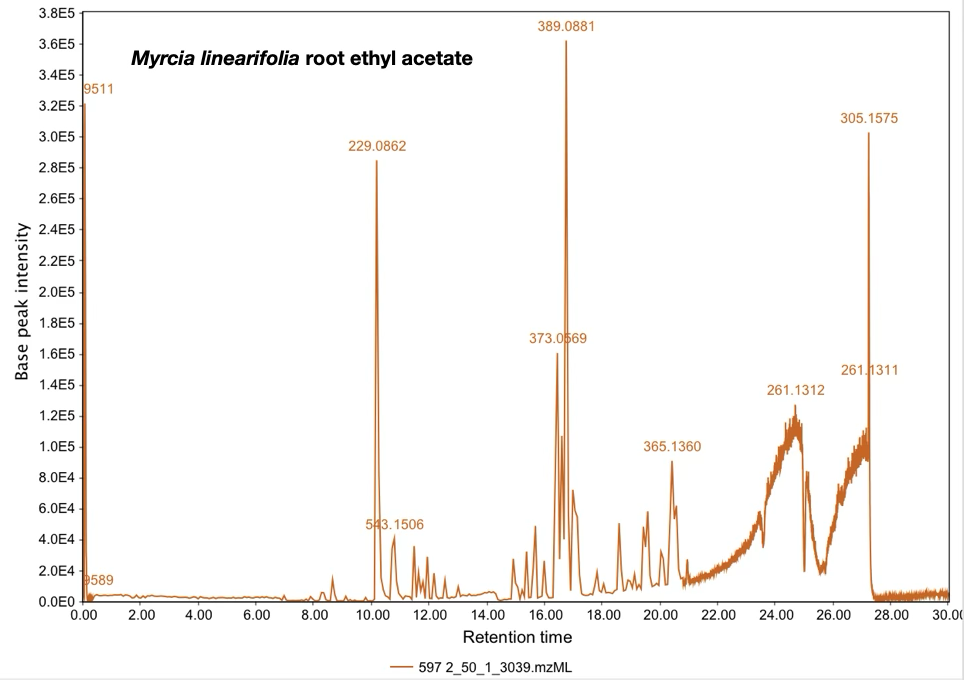


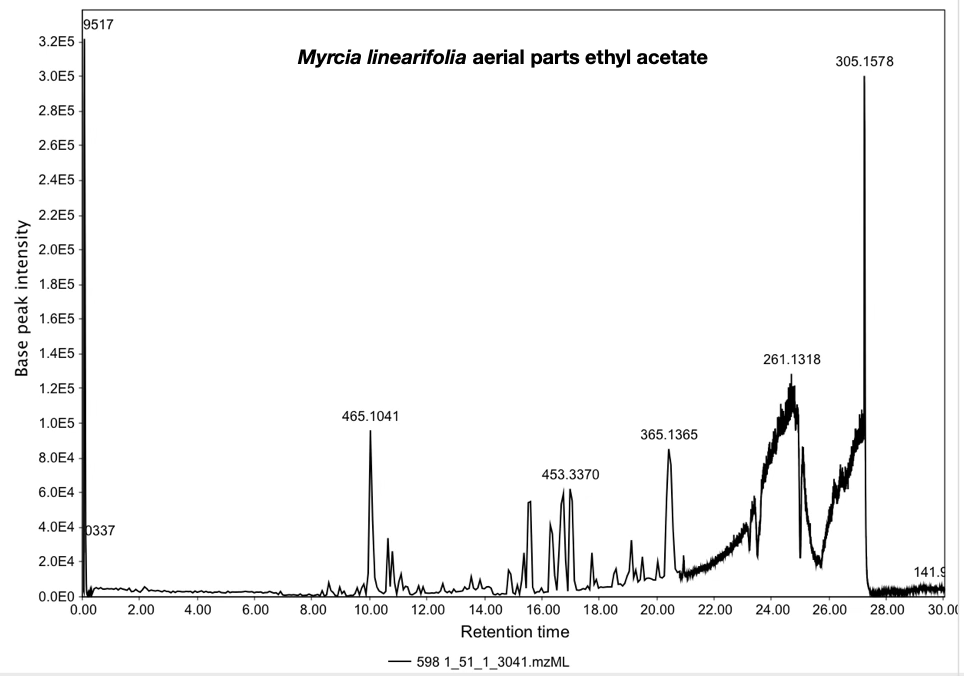


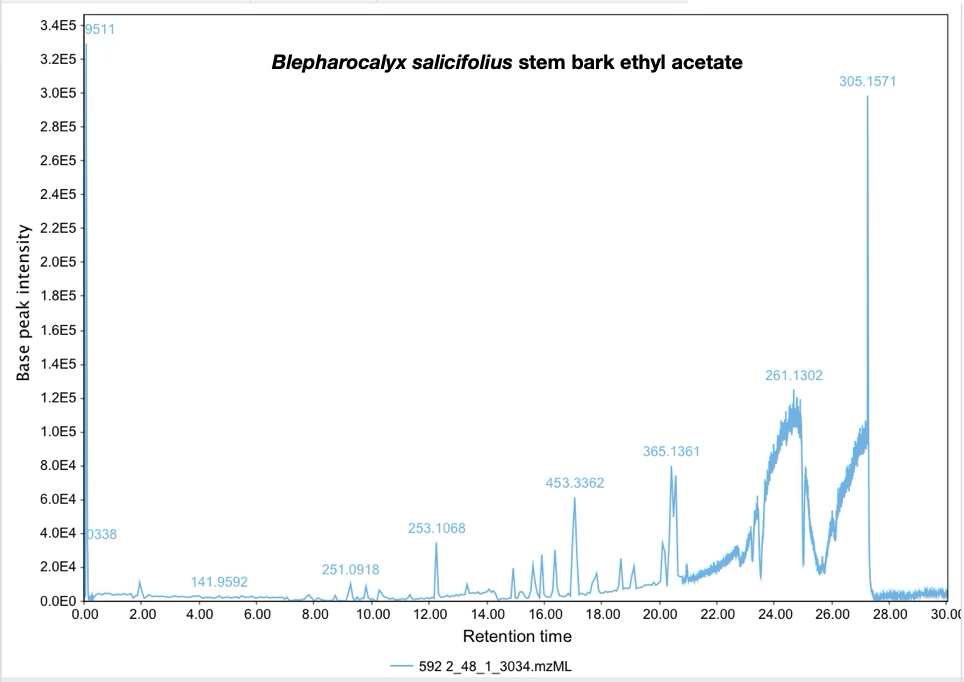


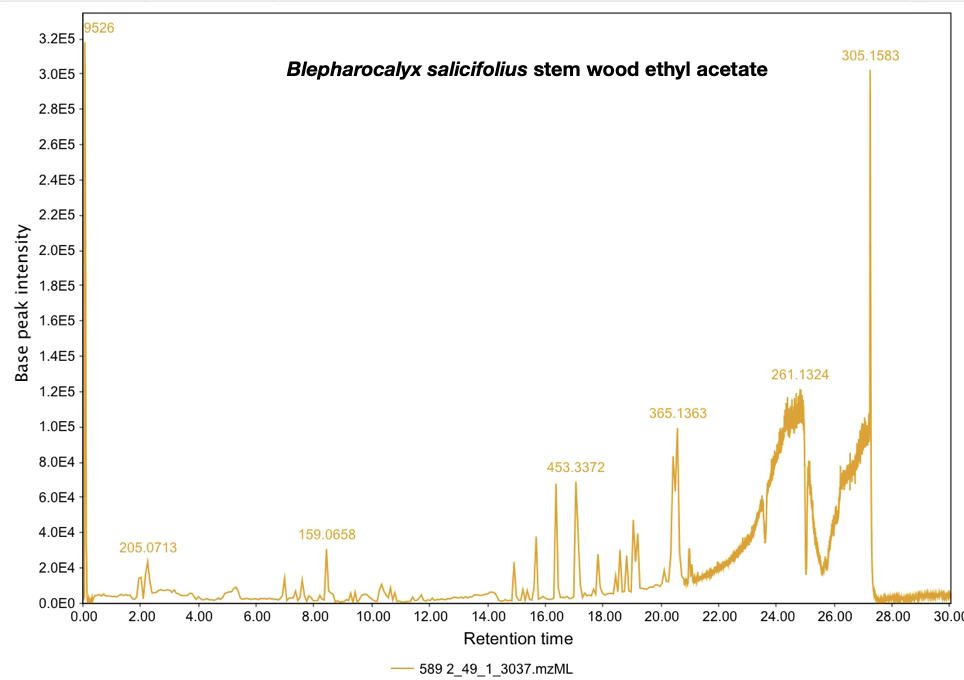


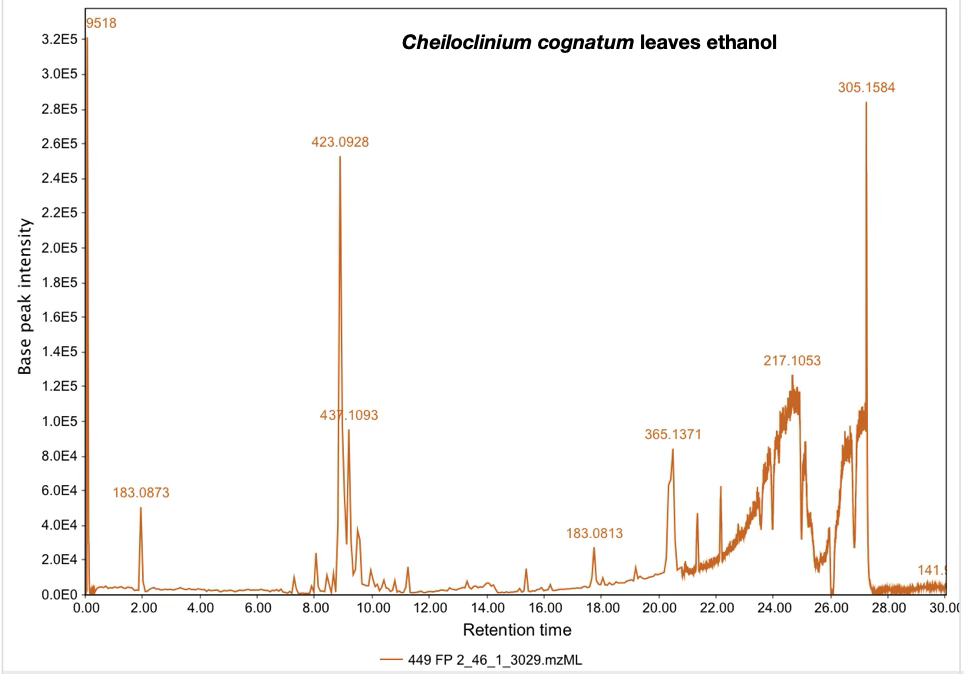


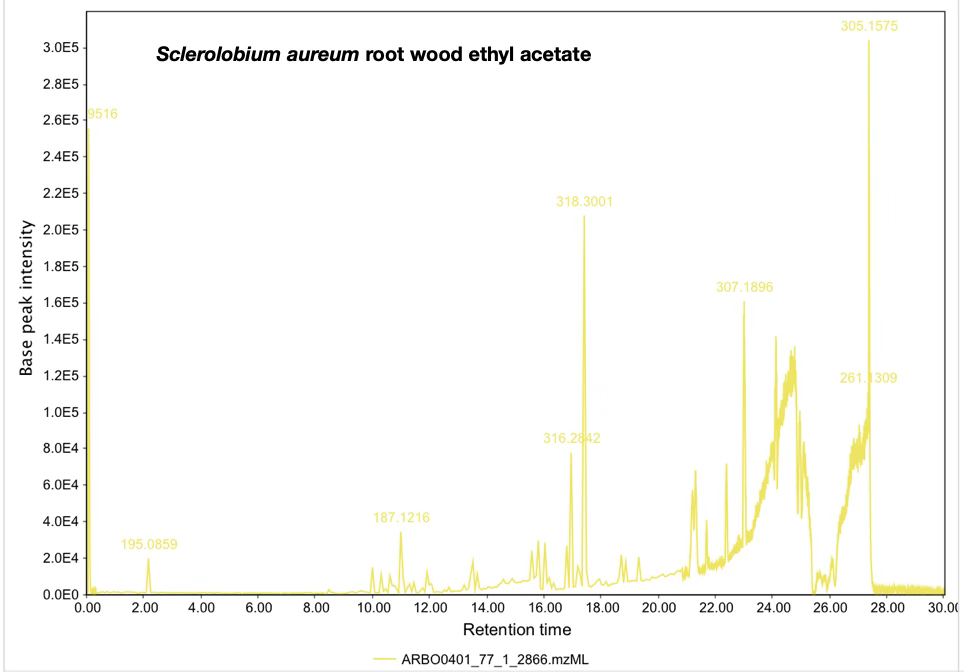


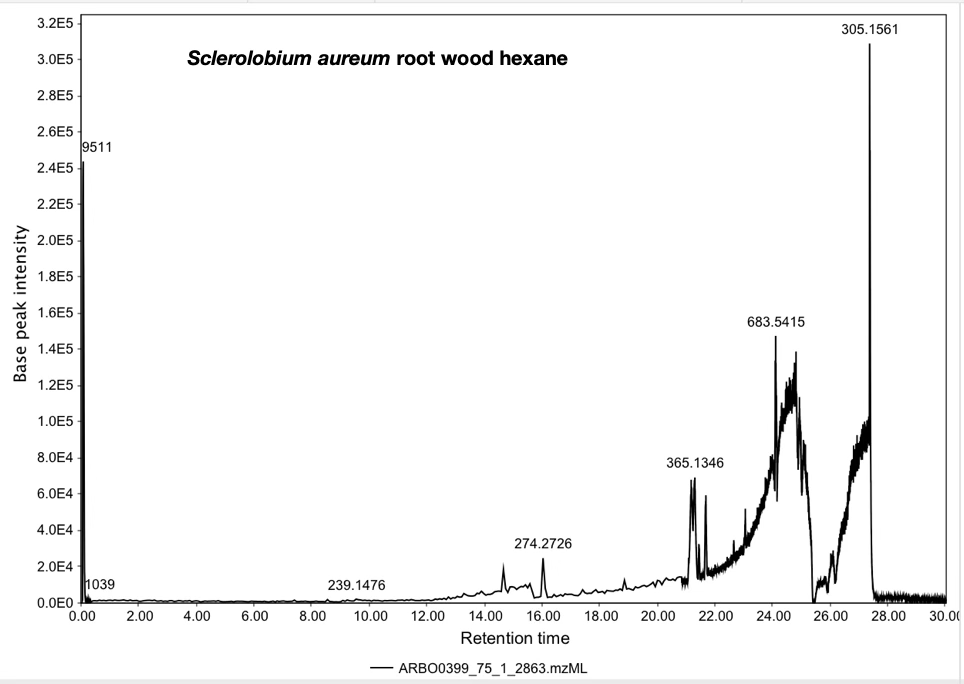


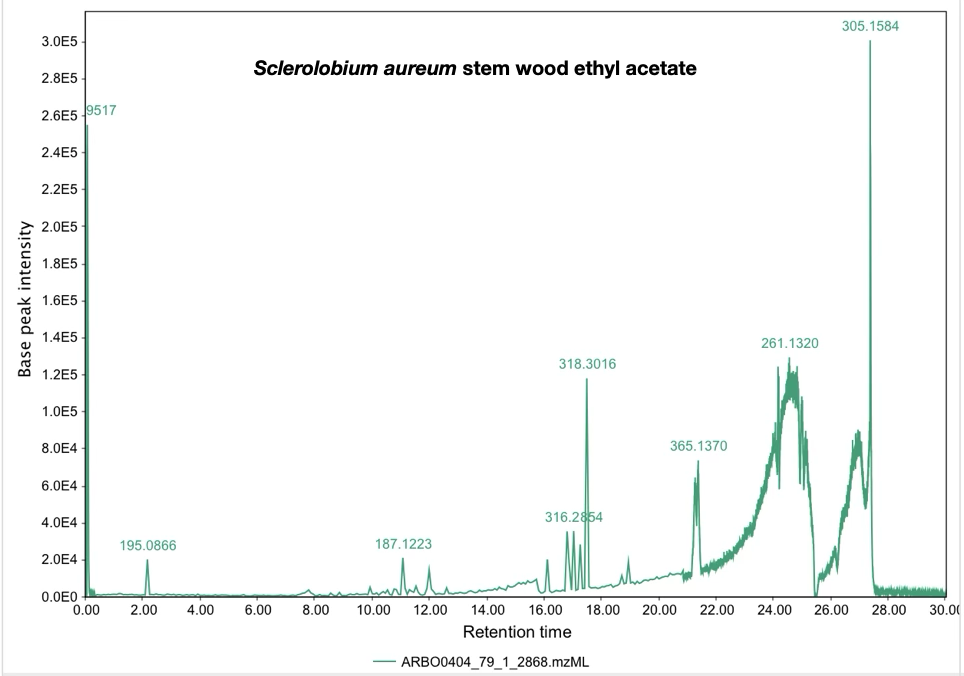


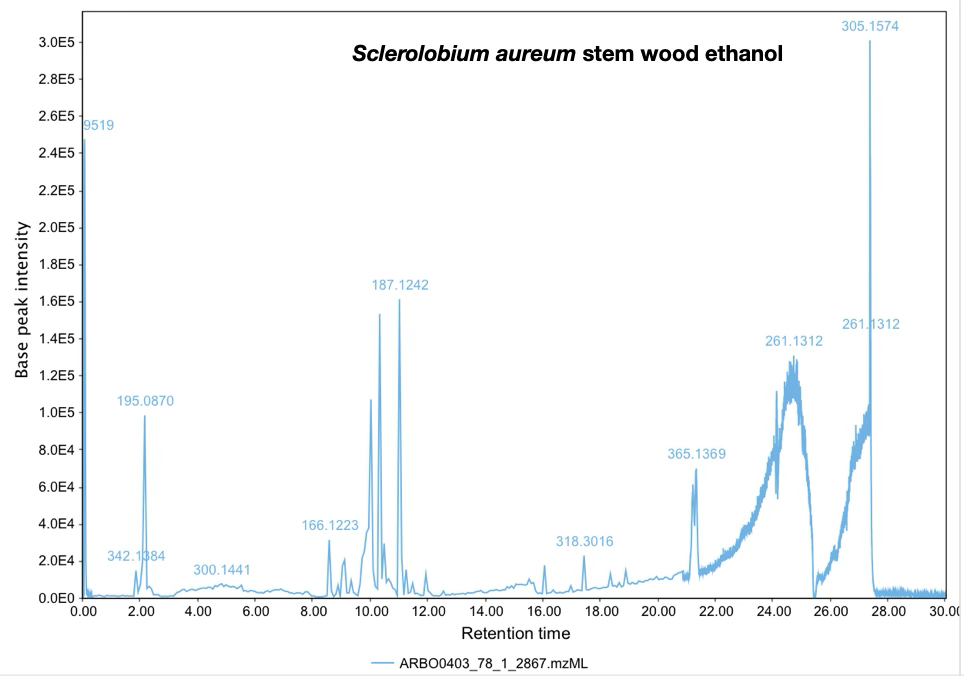


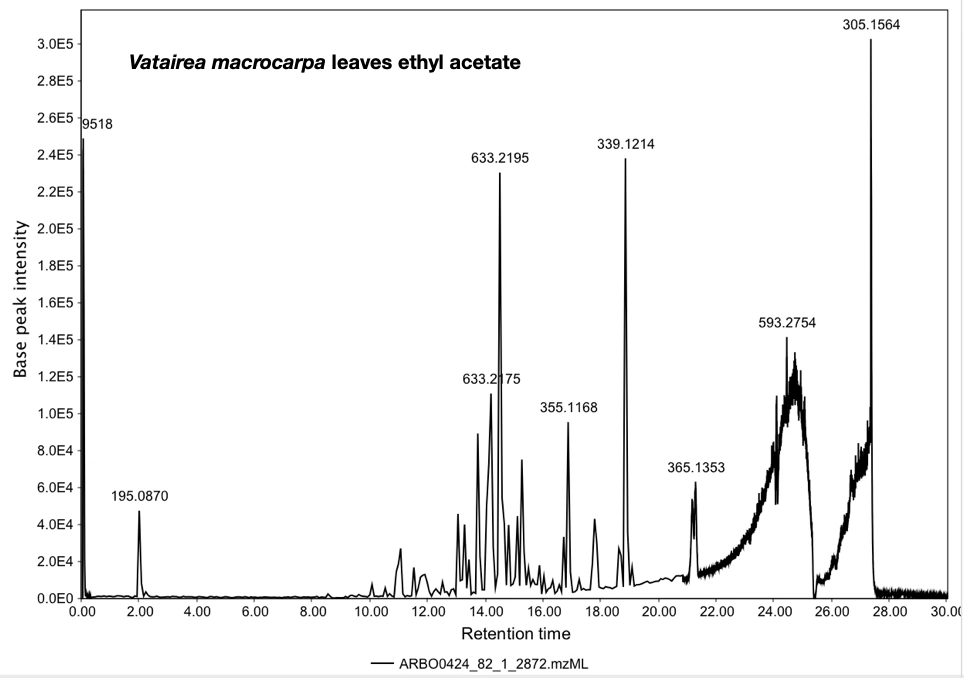


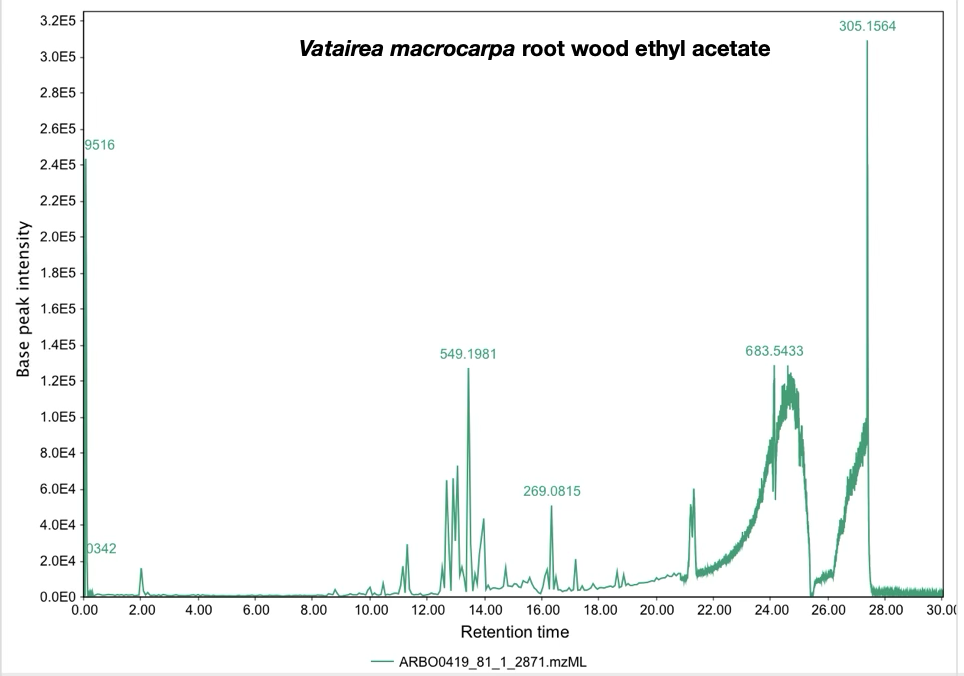


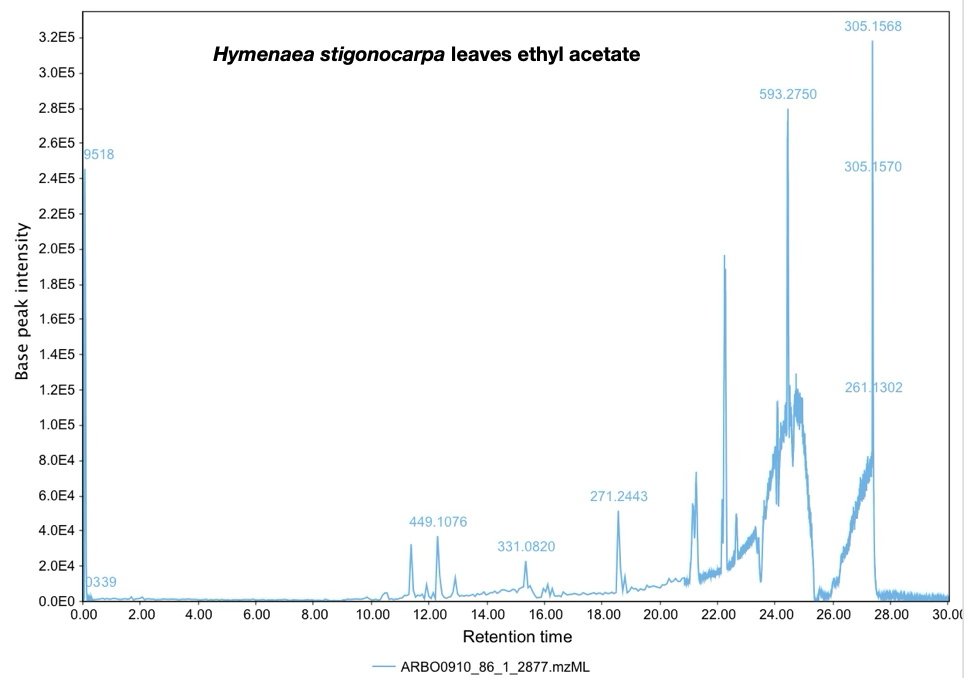


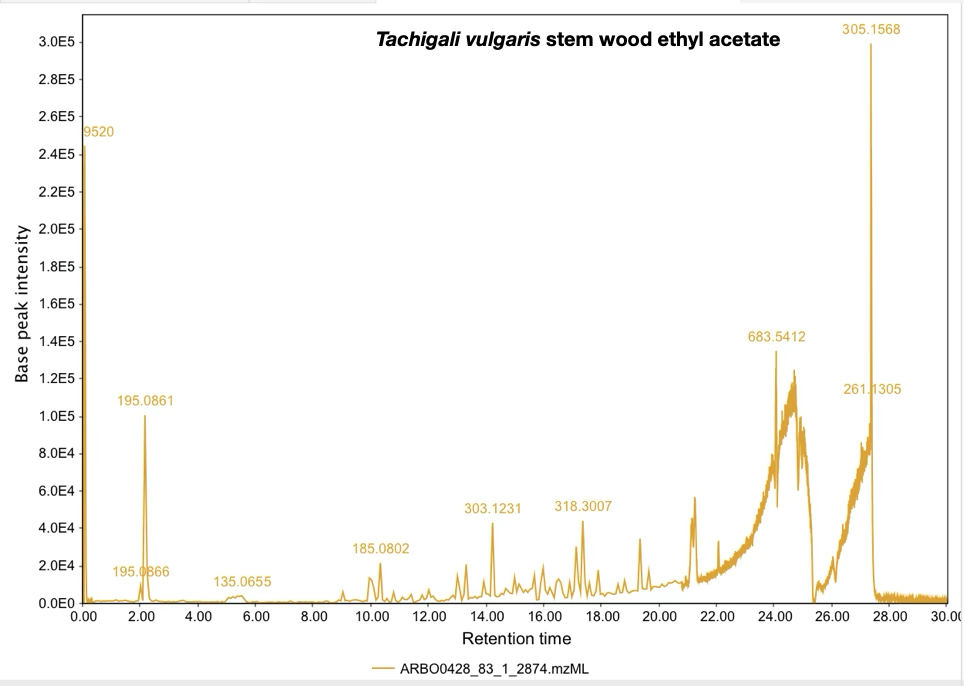

Supplement: Supplementary file 2 — Supporting File 2: cbdv71532‐sup‐0002‐FigureSI1.docx [file CBDV-23-e71532-s002.docx]
